# Solving the Global Opioid Crisis: Incorporating Genetic Addiction Risk Assessment with Personalized Dopaminergic Homeostatic Therapy and Awareness Integration Therapy

**Published:** 2024-06-20

**Authors:** Foojan Zeine, Nicole Jafari, David Baron, Abdalla Bowirrat, Albert Pinhasov, Brian Norling, Kathleen Carter Martinez, Mohammad Nami, Nima Manavi, Keerthy Sunder, David M. Rabin, Debasis Bagchi, Jag Khalsa, Mark S. Gold, Daniel Sipple, Mojtaba Barzegar, Jothsna Bodhanapati, Waseem Khader, Paul Carney, Catherine A. Dennen, Ashim Gupta, Igor Elman, Rajendra D. Badgaiyan, Edward J. Modestino, Panayotis K. Thanos, Colin Hanna, Thomas McLaughlin, Jean Lud Cadet, Diwanshu Soni, Eric R. Braverman, Debmalya Barh, John Giordano, Drew Edwards, J. Wesson Ashford, Marjorie C. Gondre-Lewis, Elizebeth Gilley, Kevin T. Murphy, Kai-Uwe Lewandrowski, Alireza Sharafshah, Milan Makale, Brian Fuehrlein, Kenneth Blum

**Affiliations:** 1 Awareness Integration Institute, San Clemente, USA; 2 Department of Health Science, California State University, Long Beach, USA; 3 Department of Applied Clinical Psychology, The Chicago School of Professional Psychology, Los Angeles, USA; 4 Division of Personalized Medicine, Cross-Cultural Research and Educational Institute, San Clemente, USA; 5 Center for Exercise and Sport Mental Health, Western University Health Sciences, Pomona, USA; 6 Department of Molecular Biology, Adelson School of Medicine, Ariel University, Ariel, Israel; 7 MEMS Precision Technology, Inc., Santa Barbara, USA; 8 Acies Biomedical, Inc. Santa Barbara, USA; 9 Division of General Education-Berkeley College, Paramus Campus, New Jersey, USA; 10 Chey-Wind Center for Trauma and Healing, Peru, USA; 11 Brain, Cognition, and Behavior Unit, Brain Hub Academy, Dubai, UAE; 12 College of Osteopathic Medicine, Western University of Health Sciences, Pomona, USA; 13 Department of Psychiatry, University of California, UC Riverside School of Medicine, Riverside, USA; 14 Division of Neuromodulation Research, Karma Doctors and Karma TMS, Palm Springs, USA; 15 The Board of Medicine, Pittsburgh, USA; 16 Division of Nutrigenomics, Victory Nutrition International, LLC, Bonita Springs, USA; 17 Department of Pharmaceutical Sciences, College of Pharmacy and Health Sciences, Texas Southern University, Houston, USA; 18 Department of Medicine, University of Maryland School of Medicine, Baltimore, USA; 19 Department of Psychiatry, Washington University, School of Medicine, St. Louis, USA; 20 Minnesota Institute for Pain Management, Minnesota, USA; 21 Hamad Medical Corporation, National Center for Cancer Care and Research (NCCCR), Doha, Qatar; 22 Karma Doctors, Palm Springs, USA; 23 Global Medical Detox Center, Menifee, CA, USA; 24 Division of Pediatric Neurology, University of Missouri, School of Medicine, Columbia, USA; 25 Department of Family Medicine, Jefferson Health Northeast, Philadelphia, USA; 26 Future Biologics, Lawrenceville, USA; 27 Department of Psychiatry, Harvard School of Medicine, Cambridge, USA; 28 Department of Psychiatry, Case Western University School of Medicine, The Metro Health System, Cleveland, USA; 29 Department of Psychiatry, Mt. Sinai University, Ichan School of Medicine, New York, USA; 30 Department of Psychology, Curry College, Milton, USA; 31 Behavioral Neuropharmacology and Neuroimaging Laboratory on Addictions, Research Institute on Addictions, University at Buffalo, Buffalo, USA; 32 Division of Primary Care Research, Reward Deficiency Syndrome Clinics of America, Inc. Austin, USA; 33 Molecular Neuropsychiatry Research Branch, NIH National Institute on Drug Abuse, Baltimore, USA; 34 Division of Clinical Neurological Research, The Kenneth Blum Neurogenetic and Behavioral Institute, LLC., Austin, USA; 35 Centre for Genomics and Applied Gene Technology, Institute of Integrative Omics and Applied Biotechnology, Nonakuri, Purba Medinipur, West Bengal, India; 36 JC’s Recovery and Counseling Center, Hollywood, USA; 37 Drew Edwards and Associates, Lakeview, USA; 38 Department of Psychiatry and Behavioral Sciences, Stanford University, Palo Alto, USA; 39 Department of Anatomy, Howard University College of Medicine, Washington, USA; 40 The Eli Foundation, West Palm Beach, USA; 41 Department of Radiation Oncology, University of California, San Diego, La Jolla, USA; 42 Division of Personalized Pain Therapy Research, Center for Advanced Spine Care of Southern Arizona, Tucson, USA; 43 Department of Orthopaedics, Fundación Universitaria Sanitas, Bogotá, D.C., Colombia; 44 Department of Orthopedics, Hospital Universitário Gaffrée Guinle Universidade Federal do Estado do Rio de Janeiro, Rio de Janeiro, Brazil; 45 Cellular and Molecular Research Center, School of Medicine, Guilan University of Medical Sciences, Rasht, Iran; 46 Department of Psychiatry, School of Medicine, Yale University, New Haven, USA; 47 Department of Psychiatry, University of Vermont, Burlington, USA; 48 Department of Psychiatry, Wright University Boonshoft School of Medicine, Dayton, USA; 49 Institute of Psychology, ELTE Eötvös Loránd University, Budapest, Hungary; 50 Center for Advanced Spine Care of Southern Arizona, Tucson, USA

**Keywords:** Opioid crisis, Medication assisted therapy, Synthetic opioids, Reward deficiency syndrome, Awareness integration therapy, Cognitive behavioral therapy, Mindfulness, Repetitive transcranial magnetic stimulation, H-Wave therapy, GARES, KB220, Trauma therapy

## Abstract

**Objectives:**

The opioid crisis in the last few decades has mounted to a global level, impacting all areas of socioeconomic, demographic, geographic, and cultural boundaries. Traditional treatments have not been deemed to show the degree of efficacy necessary to address the crisis. The authors of this review paper have set forth an unprecedented and in-depth look into multi-factorial determinants that have contributed to the opioid crisis becoming global and multi-faceted.

**Methods:**

For this narrative review/opinion article, we searched PsychINFO, PubMed, Google Scholar, and Web of Science databases to identify relevant articles on topics including the “opioid crisis,” “opioid mechanisms,” “genetics and epigenetics,” “neuropharmacology,” and “clinical aspects of opioid treatment and prevention.” Since this was not a systematic review the articles selected could represent unitential bias.

**Results:**

Despite some success achieved through Opioid Substitution Therapy (OST) in harm reduction, the annual mortality toll in the US alone surpasses 106,699 individuals, a figure expected to climb to 165,000 by 2025. Data from the Substance Abuse and Mental Health Services Administration’s (SAMHSA) National Survey on Drug Abuse and Health (NSDUH) reveals that approximately 21.4% of individuals in the US engaged in illicit drug use in 2020, with 40.3 million individuals aged 12 or older experiencing a Substance Use Disorder (SUD). Provisional figures from the Centers for Disease Control and Prevention (CDC) indicate a troubling 15% increase in overdose deaths in 2021, rising from 93,655 in 2020 to 107,622, with opioids accounting for roughly 80,816 of these deaths.

**Conclusions:**

We advocate reevaluating the “standard of care” and shifting towards inducing dopamine homeostasis by manipulating key neurotransmitter systems within the brain’s reward cascade. We propose a paradigm shift towards a novel “standard of care” that begins with incorporating Genetic Addiction Risk Severity (GARS) testing to assess pre-addiction risk and vulnerability to opioid-induced addiction; emphasis should be placed on inducing dopamine homeostasis through safe and non-addictive alternatives like KB220, and comprehensive treatment approaches that address psychological, spiritual, and societal aspects of addiction through Awareness Integration Therapy (AIT).

## Introduction

The ongoing “anti-opioid epidemic crisis” in the United States and globally continues to escalate, marked by a persistent rise in fatal overdoses, with opioids, particularly those adulterated with fentanyl, contributing to approximately 70% of these deaths. The current approaches, such as providing opioids to combat opioid dependence, may be ineffective and akin to administering alcohol to an alcoholic until death. Merely focusing on harm reduction is insufficient, and a holistic treatment approach is essential to address addiction’s physiological, psychological, spiritual, and societal dimensions. It is vitally important to delve into the history, root causes, mode of progression, and multi-factorial areas that are contributing to the spread and persistence of addiction and the fashion in which it has become a global pandemic. The authors of this paper carefully and meticulously explore all these areas from different disciplines and levels of expertise, offering a comprehensive look at these problems while offering effective solutions to eradicating the global addiction crisis.

### Present opioid crisis statistics

CDC data indicates that opioid-related overdose deaths in the United States surged to 80,411 in 2021, a notable increase from 68,630 the previous year, with synthetic opioids contributing to 72.9% of these fatalities. The American Surgeon General has forecasted that opioid overdoses will claim the lives of at least 165,699 individuals in the country in 2025, primarily attributed to the heightened potency of fentanyl [1].

**Figure 1:**
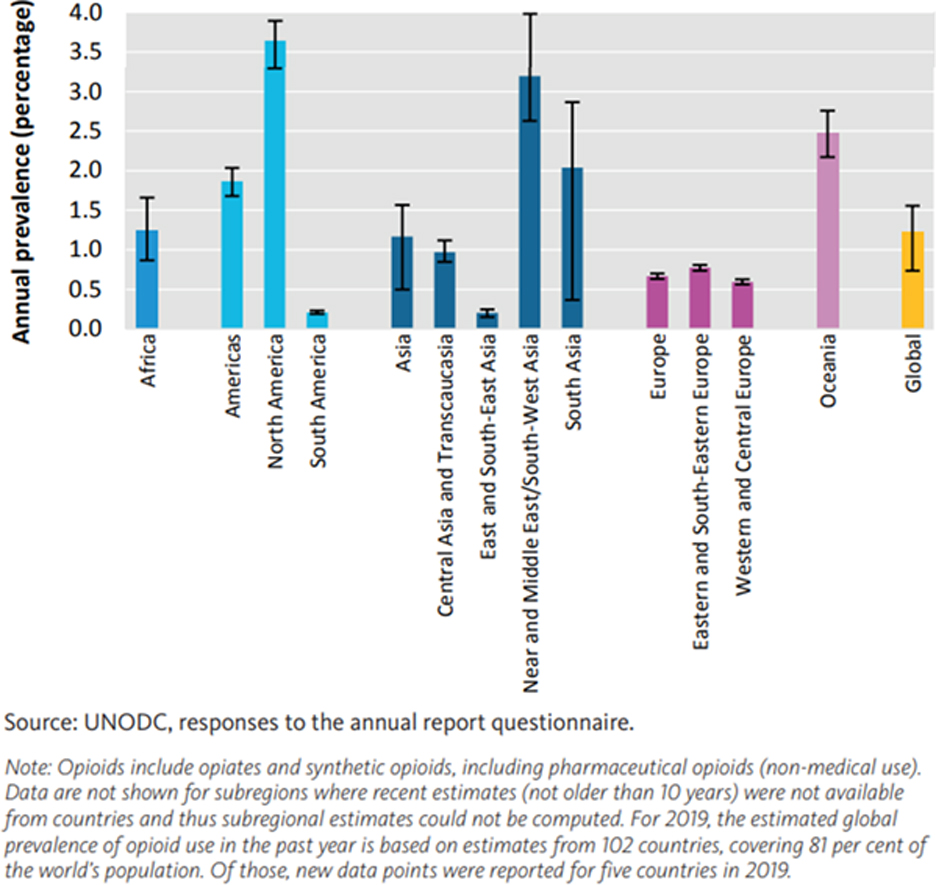
Annual Worldwide use of opioids region and subregion (2019).

The opioid addiction crisis extends beyond national borders, constituting a global epidemic with staggering societal costs. A bipartisan report by the American Medical Association (AMA) revealed that overdose deaths exact a toll of approximately $1 trillion annually in the United States. Recent statistics released on February 8, 2022, indicated that more than 106,699 individuals succumbed to overdoses in 2021 alone. Non-medical opioid use has been associated with the loss of 12.9 million Disability-Adjusted Life Years (DALYs), reflecting years of healthy life lost due to disability and premature death. From 1999 to 2019, nearly half a million individuals perished from opioid-related overdoses, encompassing both prescription and illicit opioids [2]. This grim reality is visualized in figure 1, illustrating the annual percentage increase in opioid use in population across various world regions and subregions in 2019.

In 2019, 62 million people were estimated to have used opioids (i.e., opiates and pharmaceutical and/or synthetic opioids) for non-medical reasons at the global level. This corresponds to 1.2% (range 0.7 to 1.6%) of the global population aged 15 – 64. The subregions with the highest past-year prevalence of use of opioids were North America (3.6%), the Near and Middle East/South-West Asia (3.2%), and Oceania (2.5%, essentially Australia and New Zealand). In Asia, although the prevalence of past-year opioid use is at a comparable level to the global average, more than half (58%) of the estimated global number of opioid users reside in that region. For further information, please refer to the World Drug Report published by the United Nations Office of Drugs and Crimes (UNODC) [2].

### Worldwide opioid addiction trends

**Figure 2:**
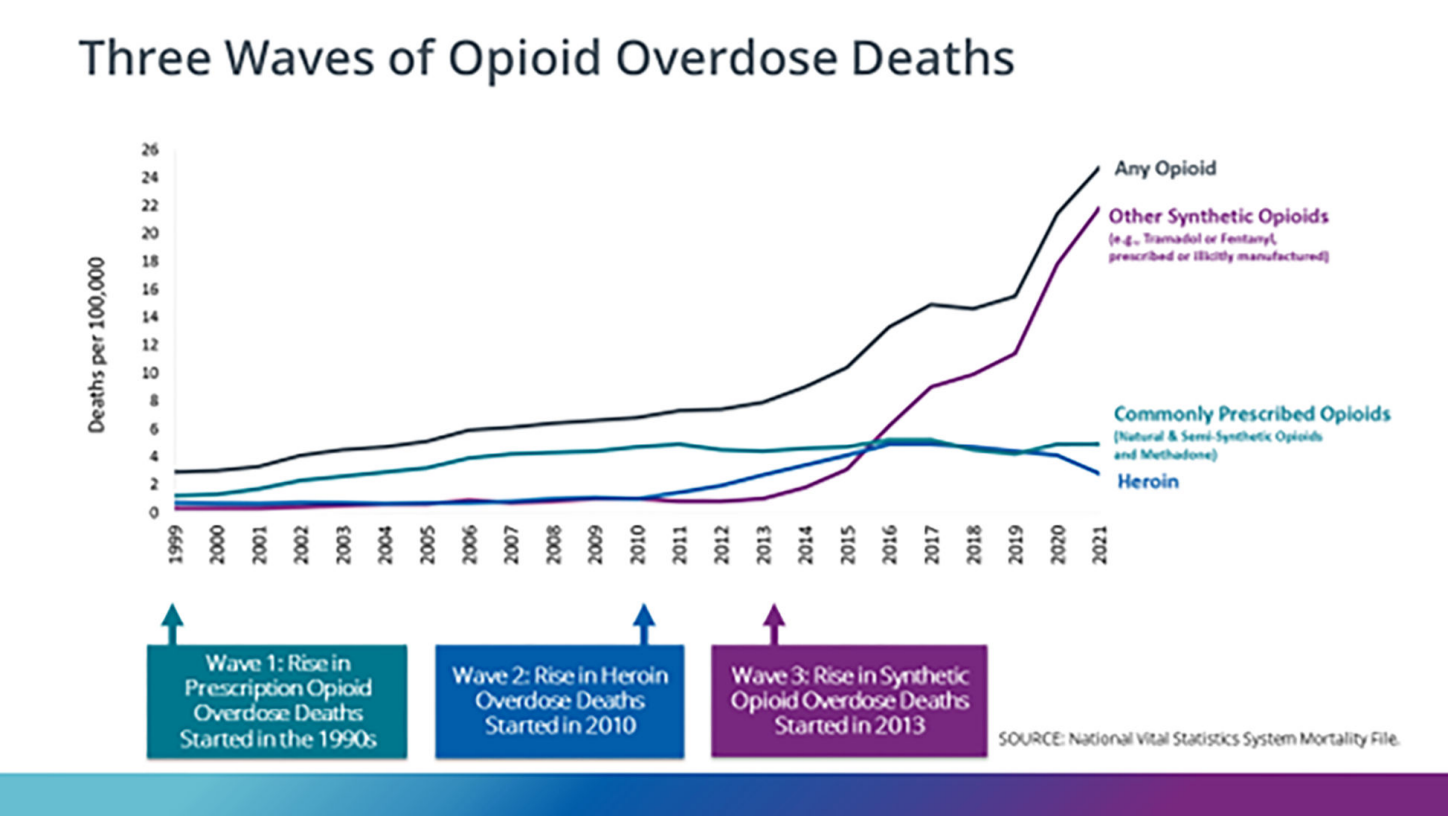
Waves of opioid compound usage evolution: from 1999–2021, nearly 645,000 people died from an overdose involving any opioid, including prescription and illicit opioids [3].

The evolution of opioid usage can be delineated into three discernible waves. Initially, the abuse of prescription opioids likely initiated the cycle of addiction for numerous individuals. Subsequently, heroin emerged as a more economical alternative to prescription opioids, followed by the introduction of synthetic opioids, which offered an even more cost-effective option. The CDC depicts this progression in opioid compound utilization in figure 2 and elucidates the corresponding trend in death rates [3].

1. The first wave commenced with a surge in opioid prescribing during the 1990s, coinciding with a steady rise in overdose deaths associated with prescription opioids (including natural and semi-synthetic opioids and methadone), which have been documented since at least 1999. Notably, the national opioid dispensing rate exhibited a decline from 2012 to 2020. By 2020, the dispensing rate had reached its lowest point in 15 years, with data indicating 43.3 prescriptions per 100 persons, totaling over 142 million opioid prescriptions.
2. The second wave emerged around 2010, characterized by a rapid escalation in overdose deaths linked to heroin. Heroin, being more cost-effective than prescription opioids, became a preferred option for individuals already ensnared in opioid addiction.
3. The third wave, initiated in 2013, marked by substantial upticks in overdose deaths involving synthetic opioids, mainly illicitly manufactured fentanyl continues to evolve, often appearing in conjunction with heroin, counterfeit pills, and cocaine [3].
4. The fourth and current wave comprises the use of fentanyl with cocaine or methamphetamine, commonly known as “speed balling” [4].

**Figure 3:**
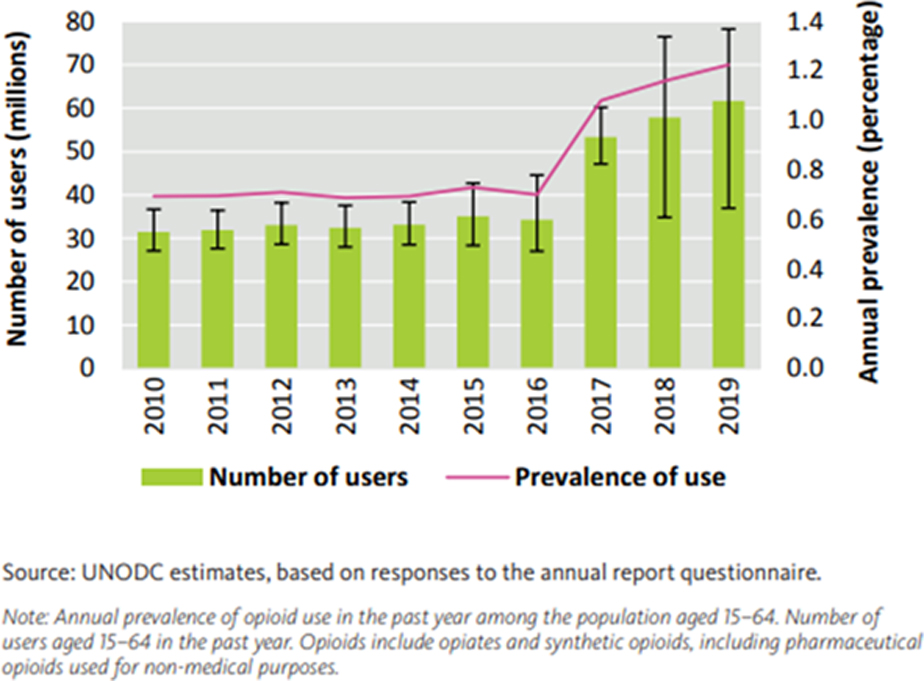
Global estimates of the number of people who use opioids and prevalence of opioid use (2010–2019). In less than a decade, the prevalence of opioid use has nearly doubled from approximately 30 million users to an estimated over 60 million [7].

Many opioid-related overdose fatalities involve multiple substances. Synthetic opioids such as tramadol and fentanyl, whether prescribed or illicitly manufactured, play a significant role in driving up the death rate, as depicted in figure 2. Notably, the potent nature of synthetic opioids disproportionately impacts users, some of whom may be unaware that they are consuming opioids [5]. The global prevalence of opioid use and the number of users is on a concerning upward trajectory, as illustrated in figure 3, underscoring the limited efficacy of current policies in the USA and abroad aimed at mitigating the opioid crisis [6].

The escalating death toll due to opioids persists, primarily attributed to the potency and ubiquity of fentanyl [8]. Notably, the incidence of fatalities associated with heroin and other commonly prescribed opioids has been on the decline in recent years. The CDC have meticulously documented the trajectory of opioid compound usage in the United States up to 2019. Since 1999, over one million individuals have succumbed to drug overdoses [9]. In 2021 alone, the United States witnessed 106,699 drug overdose fatalities. The age-adjusted rate of overdose deaths surged by 14% from 2020 (28.3 per 100,000) to 2021 (32.4 per 100,000). Of paramount concern, opioids-primarily synthetic opioids (excluding methadone)-emerge as the principal catalyst behind drug overdose fatalities. Synthetic opioids were implicated in nearly 88% of opioid-involved overdose deaths, accounting for 80,411 fatalities in 2021 (comprising 75.4% of all drug overdose deaths). Furthermore, the incidence of drug overdose deaths involving psychostimulants like methamphetamine is on the rise, both with and without synthetic opioid involvement [10]. For the first time since 2018, opioid overdose deaths decrease to 107,543 from 11,029 in 2022. Refer to table 1 for details.

### Criminalization vs Rehabilitation

A significant question, particularly pertinent to the criminal justice system, revolves around the interplay of genetics and free will. The utilization of DNA evidence in legal proceedings has become commonplace, offering potential defenses based on genetic predispositions, such as OUD. This shift underscores the intersection of genetics and the law, with DNA as a compelling source of evidence in criminal and civil cases. Legal defenses grounded in hereditary factors provide alternatives to traditional insanity pleas, aligning with contemporary genetic theories that emphasize the interplay of genes and environment in shaping behavior [12].

Historically, criminal classifications often relied on physical attributes and racial categorizations, but advances in genetic science have reshaped perceptions of criminality. Environmental determinism once dominated theories of deviant behavior, but recent genetic advancements have revived interest in biological explanations for antisocial conduct. Psychiatric genetics emerged in the 1990s, leading to the development of the G + E = P formula, which posits that behavior results from genetic and environmental factors, with genetics as the initial causal determinant [13]. In legal contexts, the question of responsibility hinges on distinguishing between free will and determinism. The legal system assumes free choice but acknowledges the influence of determinism through various defenses and mitigations. Behavioral genetic evidence has been increasingly employed in criminal defense, with recent cases leveraging genetic insights to advocate for rehabilitation over incarceration. By incorporating genetic knowledge into legal proceedings, novel precedents have emerged, emphasizing rehabilitation over punitive measures [14].

Multiple layers of dealers facilitate the distribution of illicit drugs, connecting international traffickers with end users. This process involves dealers purchasing drugs from higher-level suppliers and selling them in smaller quantities at lower market levels, completing a drug dealing “cycle” [15]. For instance, in the distribution of cocaine in Italy and Slovenia, interviews with incarcerated dealers suggest relatively organized networks with distinct market levels. The Italian heroin market, however, exhibits more “level-jumpers” who bypass certain market levels by making numerous sales per cycle, each involving smaller quantities of drugs. While data on the Slovenian market are limited, they align with these observations.

Analyzing prices enables the calculation of the revenue retained by dealers at each market level. In the Italian cocaine market, both retail sellers and international suppliers outside Italy reportedly receive approximately 30 – 40% of user spending, with the remaining 30% allocated to higher-level dealers within Italy (approximately 10% to multi-kilo dealers and 20% to lower-level wholesale dealers). These markets generate billions of euros, a substantial portion of which is laundered. Research by Caulkins and Reuter [16] suggests that less than half of revenues from established drug markets require laundering, possibly no more than a quarter. Key factors influencing this proportion include:

- Price markups across distribution levels.
- Transaction volumes.
- Participants’ cash spending capacity for daily expenses.

Police violence, a pressing public health and criminal justice concern, disproportionately affects people who inject drugs (PWID) and necessitates further investigation. Studies indicate that male gender, homelessness, arrest, drug paraphernalia confiscation, and syringe sharing are independently associated with police violence. Individuals who know someone subjected to police violence are more likely to engage in drug selling, experience arrest, and attend syringe services programs. Disparities in police violence exposure among PWID populations underscore the need to address its impact on harm reduction strategies aimed at preventing HIV and HCV transmission [17]. Scientific research has uncovered a correlation between violent and aggressive behaviors and addictions such as OUD and Alcohol Use Disorder (AUD). Genes implicated in the reward system, particularly the BRC, are linked to various addictions as well as impulsive, aggressive, and violent behaviors [18, 19].

In recent years, amidst frustration over the perceived failure in the “war against drugs,” scientists, clinicians, and policymakers have contemplated the possibility of legally prescribing all drugs of abuse, including opioids, as lifelong pharmaceutical treatments for individuals at high genetic risk, akin to managing chronic conditions like diabetes [20]. Notably, in 1973, New York Governor Nelson Rockefeller responded to escalating heroin use by abandoning lenient treatment programs and implementing the most punitive drug policy in the United States. The “Rockefeller Drug Laws” mandated severe sentences, including life imprisonment, for narcotics-related offenses, setting a precedent for subsequent “War on Drugs” policies nationwide [21]. Critics raised significant concerns about the punitive approach, arguing that it undermined liberal treatment initiatives and disregarded expert advice, transforming the welfare state into a harsh, authoritarian apparatus for societal order enforcement. These increasingly punitive measures marginalized drug users, casting them as outsiders to society and challenging their status as full citizens. The Rockefeller Drug Laws had profound repercussions for drug offenders and catalyzed a significant reevaluation of the state’s role and obligations.

As part of the National Treatment Outcome Research Study (NTORS) investigation of criminal convictions, out of 1075 clients, 54 were admitted to drug misuse treatment services across England [22]. Data were collected from the Home Office Offenders (HOO) Index at multiple time points: one year, two years, and five years after treatment intake, as well as during the year preceding the commencement of treatment. The HOO Index is a national database encompassing all convictions in adult and youth courts. The findings showed that 34% of the sample population during the year prior to treatment had been convicted of at least one offense, with significantly lower rates of convictions at the follow-up intakes.

The mean number of convicted offenses also showed statistically significant reductions in the mean number of convicted offenses. The areas of reductions in convictions were found to be acquisitive, drug-selling, and violent crimes. The findings showed that reductions in crime were associated with reductions in regular heroin use, age, and stable housing. The observed reductions in crime among drug misusers after treatment represent substantial changes in behavior and have considerable personal, social, and clinical significance. As a result, society benefited economically from these rehabilitation efforts, reduced criminality, and provided substantial economic benefits to society [22].

### Prescribing controlled substances

**Figure 4:**
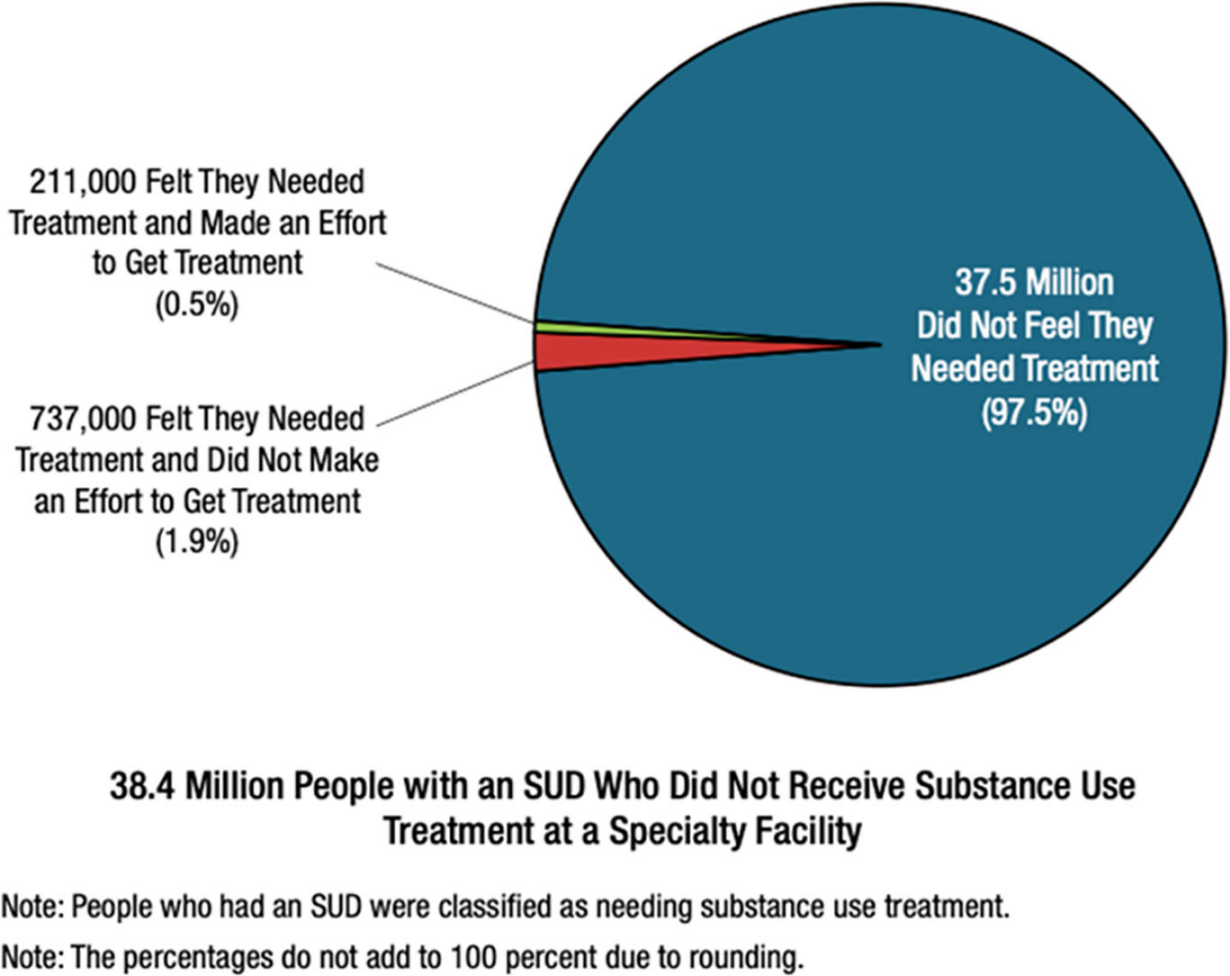
Perceived need for substance uses treatment among individuals aged 12 or older with a past year (SUD) who did not receive treatment at a specialty facility – 2020 findings [25].

This legal practice presents a significant challenge for healthcare providers, as distinguishing between legitimate medical use and potential misuse is complex [22]. Historical instances have highlighted the dangers, with some physicians prescribing potent opioids to known abusers of painkillers, facing threats of violence when refusing to continue providing access to controlled substances. Tragically, this has resulted in fatalities, such as the case of Stephen Pollock, a San Antonio-based MD and researcher involved in morphine detection [23]. In the 1990s, efforts to address the chronic undertreatment of severe pain led to the expanded prescribing of opioid analgesics. However, this approach inadvertently fueled overuse, diversion, OUD, and overdose [24]. This dilemma presents a “Catch-22” scenario: healthcare providers must balance between undertreating pain, causing unnecessary suffering, and overtreatment, risking adverse outcomes like increased OUD and overdose. Although opioid analgesic prescribing peaked in 2011 and has since declined, concerns persist about the ongoing opioid crisis in both public discourse and scientific literature. Given the opioid epidemic, prescribing opioid analgesics for chronic pain is now contentious and lacks definitive standards. Healthcare providers must prioritize appropriate patient assessment and treatment planning to navigate these complexities effectively figure 4.

Given the high rate (97.5%) of individuals resisting treatment, a critical question arises: is an opioid deficiency linked to poor abuse. As a function of prefrontal cortical unmyelinated sites in our youth, a gateway to future opioid abuse? We wonder what if any are the profound effects of early use of opioids have on brain development as an epigenetic insult that carries forward in adulthood.

### Multi-factorial causality of addiction

Every day, millions worldwide struggle to break free from their dangerous and often deadly attraction to intoxication; for some, this pursuit of a “high” may equate to seeking happiness. Among the most vulnerable to substance abuse are adolescents, whose brains are still undergoing significant development and maturation. The transition from childhood to adolescence brings about profound cognitive changes, impacting decision-making abilities and leading to risk-taking behaviors, a sense of invincibility, and impulsive actions. Epigenetic alterations involving chemical modifications to DNA and histones play a critical role in neurodevelopment [26, 27]. The consensus in the literature suggests that early life experiences, such as maternal nurturing coupled with genetic factors contribute to shaping an individual’s social characteristics. Salo et al. [28] demonstrated that strong maternal nurturance during childhood predicted high reward dependency and reduced avoidant attachment in carriers of the serotonin receptor gene (HTR2A) T/T genotype but not in those with the T/C or C/C genotype. However, the temperament and character inventory findings did not fully support this conclusion [29–32].

Recently, Levey et al. [33] conducted a transcriptome-wide association study analysis and identified significant associations with the expression of NEGR1 in the hypothalamus and DRD2 in the NAc, among others, in approximately 1.2 million veterans and 59,000 African Americans with significant depression. This finding is significant because original research by Blum et al. [34] revealed that the DRD2 A1 allele was not specific to alcoholism but rather linked to a nonspecific reward phenotype such as depression/anhedonia.

The consequences of these epigenetic structures can be observed in an individual’s behavior and characteristics, underscoring the significance of gene-environment interaction, particularly concerning psychoactive drugs [35–46].

Genomic imprinting, a mechanism whereby DNA methylation silences or deactivates genes, involves the selective silencing of one allele inherited from either parent, leading to imprinted genes [47]. These parent-of-origin effects may be transmitted through gametes to offspring, contributing to various disorders like Angelman Syndrome and Prader-Willi Syndrome [48]. This underscores DNA methylation’s role in major psychiatric disorders such as schizophrenia, bipolar disorder, major depressive disorder, autism, and related conditions. Understanding the aberrations in this mechanism offers insights into the etiology of these disorders and suggests the potential utility of DNA methylation as a therapeutic target [49–59].

### Early experiences

Childhood abuse, encompassing both abuse and neglect, stands out as a significant risk factor for adolescent substance use. However, it’s crucial to emphasize that not all families engage in abusive behavior. The Federal Child Abuse Prevention and Treatment Act (CAPTA) defines abuse as actions or omissions by a child’s caregiver that lead to physical or psychological harm [60]. For example, one study reported that 29% of abused children were involved in some degree of substance use, while another found that 16% of abused children engaged in drug use [61]. These findings underscore the detrimental impact of childhood abuse on the development of healthy behaviors and highlight the urgent need for effective interventions to support at-risk children and families.

The environment interacts with the human genome, facilitating crosstalk between the genome and its surrounding environment. This interaction can modify adverse epigenetic states stemming from events or exposures during earlier life stages [62]. Structural abnormalities associated with early adverse experiences are frequently observed in brain regions responsible for regulating and modulating emotionality, establishing a direct connection between childhood adversity and psychopathological behavior in adulthood [63, 64]. It is imperative to comprehend the nutritional programming of epigenetic states, the longevity of these marks over time, and their impact on biological function and health across current and future generations [31, 65, 66].

### Transition to adolescence

When considering the emergence of risk factors, it is crucial to recognize the impact on cognitive development in the context of adolescent substance use. Adolescence, a complex developmental stage, marks a period of significant brain development, rendering individuals particularly susceptible to stress and risk-taking behaviors as they expand their social networks and experiment with various forms of social interaction [67, 68].

Stressful circumstances, including familial and social tensions, as well as experiences of abuse during critical developmental stages, can heighten responsiveness to addictive substances and increase the propensity for developing a SUD. As preteens approach adolescence, they may need to prepare to make wise decisions since their brains are not fully developed or myelinated like those of developed adults. The midbrain region, which governs social and emotional behavior, is particularly vulnerable to dysfunctions that can lead to deficits in neurotransmitter production. This vulnerability extends to the Pre Frontal Cortex (PFC), commonly referred to as the “braking/inhibitory system,” which plays a critical role in executive function and decision-making. The subcortical areas of the midbrain can override the PFC, underscoring the importance of understanding how stress and substance abuse, such as alcohol and cocaine, can alter white and gray matter volume integrity [69].

Caulkins et al. [15], Blum et al. [69 ], Fowler et al. [70], Blum et al. [71] independently investigated the relationship between genotypes and homophily (friendships). They found that individuals from families with SUD, or RDS families, had a higher prevalence of the DRD2 Taq1 and DAT1 10/10 alleles compared to controls (p < 0.015). Interestingly, the presence of the TaqA1 allele in every single family member suggests the existence of homophily among individuals carrying the A1 variant. Humans, particularly teenagers, tend to associate with individuals who share similar traits, but it remains unknown whether this propensity influences genotype distribution in a population. Blum et al. [69] investigated this phenomenon and found that certain genotypes, such as DRD2, exhibited a positive correlation with homophily, while others, like CYP2A6, showed a negative correlation (heterophily).

Puberty heralds a period of brain development characterized by distinct changes, including decreased gray matter, increased white matter, and augmented dopaminergic connections that facilitate the maturation of the PFC and limbic system [72]. Gray matter comprises neuronal cells in the brain specialized for specific functions. In contrast, white matter relays information from sensory organs to the cerebral cortex, encompassing axons involved in emotional and hormonal functions [72]. Gender differences in brain development have been noted, particularly among adolescent males, who exhibit significant reductions in gray matter and increases in white matter during puberty [73]. These disparities may stem from the influence of sex hormones, which are released in heightened concentrations during this developmental phase [74, 75], alongside recognized DNA predispositions to addiction risk.

It is well recognized that the PFC begins to mature before an individual reaches their early 20s when myelination first begins [76–79]. Stress and/or medications during this developmental phase can impair myelination, which is essential for controlling brain speed, and may be particularly vulnerable during pre-developmental phases [80, 81]. Individuals face numerous difficult questions and experience dissatisfaction during the challenging years before adulthood, and these experiences, along with potential genetic and epigenetic hazards, may serve as precursors to later drug dependence [82]. The risk-taking, invincible, and whimsical brain states characteristic of teens may contribute to their propensity to experiment with drugs and addictive substances [83]. Additionally, several other factors may contribute to adolescents’ vulnerability to addictive behavior. The multifactorial causes contributing to an increased risk of drug use among adolescents include social factors, family dynamics, deviant peer relationships, social status, bullying, and gang affiliation. Social and family influences often coexist, creating a complex interplay of risk factors that predict adolescent drug use [83].

### Neurogenetics of opioid addiction causality

Despite extensive investigations, particularly in the preclinical setting, the pathophysiology and etiology of addiction or RDS remain poorly understood. While 102 publications are listed under the phrase “neurogenetics of opioids,” the broader term “genetics and opioids” yields 11,300 papers. This discrepancy highlights a gap between preclinical models of addiction and the clinical criteria for the condition outlined by DSM-5, as noted in a recent analysis by Belin-Rauscent et al. [84]. As part of an ongoing recovery strategy, Gold et al. [85] recommended the continued use of “magic bullets,” such as clonidine and possibly buprenorphine, to counteract the “opiate drive state.” The aim is to restore the brain’s homeostasis to an opioid/opioid-free, sober state while preserving the necessary drive for creative solutions to achieve and maintain a joyful existence.

In this context, Blum initially formulated the “endorphin deficit theory” for alcohol and opiates in his early work and collaborations with others [86–88]. Building on this, Gold et al. [89, 90] proposed that endorphin insufficiency, possibly hereditary, may precede opiate usage in individuals with addiction. They also suggested a role for dopamine in opiate withdrawal and proposed that the use of potent exogenous endorphinomimetic substances could lead to endorphin abnormalities [91]. Furthermore, Gold et al. [92] posited that the normal inhibitory tone at the locus coeruleus and during opiate withdrawal is provided by endogenous peptides physiologically, and the attenuation of this inhibitory mechanism due to decreased endogenous peptides results in norepinephrine-induced hyperactivity.

Earlier research by Blum and colleagues suggested similarities in the mechanisms of alcohol and opiate withdrawal [93–97], potentially due to isoquinoline’s opiate-like effects, which provided insights into the role of dopamine in acute opiate abstinence [74]. Notably, chronic morphine dependence can lead to a prolonged withdrawal period upon abstinence. Kaufling and Aston-Jones [98] have demonstrated adaptations in rodent models involving Ventral Tegmental Area (VTA) dopamine neurons.

Opiate withdrawal induces behavioral changes attributed to altered reactivity of mesolimbic dopaminergic neurons, dopamine cell death, and other adaptations. Kaufling and Aston-Jones [98] also highlighted the importance of the Rostromedial Tegmental Nucleus, a cluster of GABAergic neurons in the VTA (tVTA) tail, in modulating opiate behavioral responses. After two weeks of abstinence, they found that VTA dopamine neurons, but not tVTA GABAergic neurons, develop tolerance to morphine. Moreover, optogenetic activation of tVTA neurons suppressed VTA dopamine neurons in both opiate-naive and chronically withdrawn rats. Interestingly, tVTA inactivation enhanced VTA dopamine activity in drug-naive rats but not in those previously exposed to opiates, mimicking the effects of opiates on dopamine cells. This suggests that prolonged withdrawal impairs tVTA’s ability to exert disinhibitory control over dopamine neurons even while preserving inhibitory control.

Furthermore, recent studies [67] have revealed that morphine withdrawal reduces both tonic glutamatergic input to VTA dopamine neurons and tVTA neuronal activity. This finding indicates that alterations in glutamate and GABA feedback contribute to the apparent tolerance of VTA dopamine neurons to opiates after prolonged exposure. It is important to note that long-term abstinence from drugs like morphine leads to tVTA inhibition rather than disinhibition.

Chronic opiate exposure may amplify negative feelings during withdrawal by impacting dopamine cells, resulting in decreased dopamine levels and a need to restore “dopamine homeostasis,” particularly in individuals carrying the DRD2A1 gene [34, 99]. It is widely recognized that addictive substances act by stimulating the mesolimbic dopamine pathway, where they modulate synaptic plasticity mechanisms such as long-term potentiation or depression. Additionally, they induce gene transcription and function changes, partly mediated by epigenetic adaptations [100–109] and various neuroadaptations between systems [100, 101].

These modifications influence stress response and inhibitory control through glutaminergic/GABA processes in brain regions such as the NAc [110], amygdala [111], dorsal striatum (DS) [112], PFC [113], and among others. Since the late 1960s and early 1970s, research has been dedicated to understanding relapse and drug resumption. One fundamental principle posits that a single “psychological extinction” method can effectively reduce recurrence rates by eliminating the motivational factors driving drug use and diminishing the drug’s reinforcing effects. It is widely recognized that various biological mechanisms can attenuate the immediate effects of dopamine, thereby reducing the urge to use it.

Over the past decade, there has been significant global interest in preclinical models that address psychological constructs and clinical criteria associated with addiction outlined in the DSM-5 and earlier versions. These models have focused on elucidating mechanisms such as receptor blockade, transporter inhibition, low blood-brain barrier penetration, and epigenetic regulation of gene expression, among others, involved in biosynthesis, storage, catabolism, and neuronal release. Notably, specific preclinical models aimed at understanding the return to “reward deficit” over prolonged periods, particularly in the context of opiate/opioid abstinence, have evolved and are favored by Medication-Assisted Treatments (MATs) approved by the FDA [6, 114, 115]. The persistent seeking behaviors observed in heroin users are influenced by environmental factors, possibly including epigenetic factors, and can progress to compulsive behaviors. Addiction, due to hereditary reward gene polymorphisms, may persist throughout life following sustained drug exposure, particularly in genetically predisposed individuals [116].

In line with this, Zou et al. [117] utilized functional magnetic resonance imaging (fMRI) to demonstrate weaker connections between reward-processing brain regions and areas related to motor skills in 30 heroin-dependent subjects after three years of abstinence compared to healthy controls. Some participants showed signs of recovery, reducing the risk of relapse. This underscores the potential for brain recovery from heroin-induced injury and suggests that treatment should be maintained for at least three years. However, genetic reward gene polymorphisms were not assessed in Zou et al.’s study [117]. Formal genetic testing could aid clinicians in determining appropriate treatment duration, as heroin addiction treatment should extend beyond detoxification to address underlying genetic vulnerabilities and provide long-term neuroscience-based interventions. Many adolescents and adults in America today are enrolled in self-help programs with a high risk of relapse due to undiagnosed genetic polymorphisms, highlighting the need for comprehensive long-term treatment [5].

Future preclinical models should focus on identifying the pathophysiological bases of addiction and associated vulnerability endophenotypes. Utilizing contemporary neuroimaging tools and studying hereditary risk factors, including epigenetic modifications, will enable the clinical community to provide better care for individuals addicted to opiates/opioids. An effective translational approach integrating cognitive neuroscience, animal research, and correlational techniques in humans, such as genome-wide and candidate polymorphism analysis, is necessary to elucidate the complex etiology of addiction and guide targeted interventions [118].

According to specific theories, an addicted endopheno-type may not be specific to the substance of choice but may generalize to altered brain reward circuits affecting net mesocorticolimbic dopamine release. Gondre-Lewis et al. [119] proposed that dopaminergic reward circuits can be genetically or epigenetically altered, potentially leading to uncontrollable drug intake. For example, decreased dopamine D3 receptor (DRD3) availability has been associated with increased sensitivity to opioids. Expanding on this concept, Gondre-Lewis et al. conducted a comprehensive polymorphism risk analysis in a human cohort of chronic opioid users, revealing a higher frequency of the polymorphic DRD3 risk allele (rs6280) compared to the opioid receptor 1 in African Americans (rs1799971). These findings suggest that dopamine-related receptors, particularly DRD3, may predominantly influence opioid-seeking behavior among African Americans, underscoring the importance of attention to DRD3-mediated modulation of dopaminergic homeostasis for the development of novel neuropharmacological therapies targeting OUD [119]. Furthermore, the hypocretin/orexin (HCRT) system has been implicated in both positive and negative drug reinforcement, with HCRT receptor 1 (HCRT-R1) signaling implicated in drug-related behaviors across various drug classes, including opioids. Schmeichel et al. [120] demonstrated that systemically administered NBI-80713 dose-dependently decreased heroin self-administration in animals with extended access, modeling the transition from controlled to compulsive-like drug use. Additionally, they found an increase in Hcrtr2 mRNA levels in the central amygdala, a stress-related brain region, in animals with extended heroin access, suggesting a functional role for HCRT-R2 signaling in compulsive-like heroin self-administration. These findings highlight HCRT-R2 antagonism as a potential pharmacological target for the treatment of heroin dependence.

The modulation of pain and analgesia involves the Mu-opioid receptors (MORs), representing a candidate mechanism for developing biomarkers of pain conditions and treatment responses. Comparing homozygotes with OPRM1 G carriers, studies revealed an overall reduction of baseline μ-opioid receptor availability in regions implicated in pain and affective regulation. Moreover, G carriers showed more pronounced mood disturbances, lower placebo-induced μ-opioid system activation in several brain regions, and lower levels of dopamine D2/3 activation in the NAc. Additionally, G carriers reported higher NEO-Neuroticism scores at the trait level. This indicates that the A118G OPRM1 polymorphism contributes to interindividual variations in neurotransmitter function responsive to pain and highlights its role in modulating pain perception and placebo responses [121].

The introduction of depressive symptoms, including social withdrawal, is considered a leading cause of relapse. Lutz et al. [122] demonstrated that delta and kappa opioid receptor (DOR and KOR, respectively) knockout mice develop more substantial or reduced emotional disruption during heroin abstinence, establishing DOR and KOR activities as protective and vulnerability factors, respectively, that regulate the severity of abstinence. Moreover, these investigators found that chronic treatment with the antidepressant fluoxetine prevents the emergence of low sociability, with no impact on the working memory deficit, implicating serotonergic mechanisms predominantly in the emotional aspects of abstinence symptoms. Finally, targeting the main serotonergic brain structure, Lutz et al. [122] show that gene knockout of MOPRs in the dorsal raphe nucleus (DRN) before heroin exposure abolishes the development of social withdrawal. These findings help us understand opioid system-mediated serotonin homeostasis in heroin dependence.

Aldehyde dehydrogenase 1A1 (ALDH1A1), a retinoic acid (RA) synthase, is selectively expressed by the nigrostriatal dopaminergic (nDA) neurons that preferentially degenerate in Parkinson’s disease (PD). ALDH1A1-positive axons mainly project to the DS. Pan et al. [123] found that μ-type opioid receptor (MOR1) levels were severely decreased in the DS of postnatal and adult Aldh1a1 knockout mice. In contrast, the dietary supplement of RA restores its expression. Furthermore, RA treatment also upregulates striatal MOR1 levels and signaling.

Pharmacological and electrophysiological evidence has shown that opioid receptors are involved in the mechanism of heroin dependence. The MOR, OPRM1, has long been a high-priority candidate for human genetic studies of addiction. The G allele showed a modest protective effect on general substance dependence (OR = 0.90, 95% C.I. [0.83 – 0.97], p-value = 0.0095, N = 16,908 [124]. The stress axis is regulated, in part, by the endogenous opioid beta-endorphin, acting on MORs. Individuals carrying one or two copies of the G allele of the MOR gene (OPRM1 A118G) may have higher receptor binding for beta-endorphin compared with A.A. homozygotes, contributing to individual differences in cortisol reactivity to stress. Lovallo et al. [125] measured cortisol in 251 young adults (69 GA/GG vs 182 AA genotypes) exposed to mental arithmetic plus public speaking stress relative to a resting control day. Women had smaller cortisol responses than men (F = 10.2, p = 0.002), and women with G.A. or G.G. genotypes (N = 39) had an absence of cortisol response relative to A.A. carriers (N = 110) (F = 18.4, p < 0.0001). Male genotypes had no such difference in response (F = 0.29). Cortisol response following MOR blockade using naltrexone in 119 subjects unmasked a more significant tonic opioid inhibition of cortisol secretion in women (N = 64), consistent with their blunted stress reactivity. Compared with men, women may have cortisol stress responses that are more heavily regulated by endogenous opioid mechanisms, and the OPRM1 GA/GG genotypes may affect females differentially relative to males. Diminished cortisol responses to stress may affect health behaviors in women with GA/GG genotypes.

In summary, research demonstrates that opioid signaling regulates the net release of dopamine in the NAc in the mesolimbic area of the brain through various polymorphic genes associated with neurotransmitters and second messengers. The leading associations of these reward genes are wellness, stress-coping abilitity, incentive salience (wanting), and motivation. Notably, Carlsson, Greengard, and Kandel received the Nobel Prize in 2000 for their research on dopaminergic activity’s molecular and cellular role in neurons. Serotonin, endorphins, glutamate, and dopamine were all comprehensively described in the historical and significant psychopharmacological study by Blum et al. on the role of neurotransmitters and associated behaviors [116]. Prescribed opioids and easy availability are the primary causes of the second and deadliest opioid epidemic the United States has ever seen. Currently, the clinical consensus is to treat OUD with long-term to permanent opioid replacement treatment, treating it as though it were an opioid deficiency condition. Similar to how insulin is used to treat diabetes, opioid agonist administration has been considered by some to be essential to make up for lost opioids, treat OUD, and prevent overdoses. In that opioid, clinicians believe that agonist MATs are seen as a crucial component of therapy, OUD and addiction treatment are conceptualized similarly to endocrinopathy [125]. Is this strategy rational? Is the long-term use of opioids to treat OUD advantageous or detrimental, aside from harm reduction?

### Stages of opioid intake

It is essential to acknowledge the critical role of brain regions within the mesolimbic system in opioid-induced neuroplastic adaptations, including the basal ganglia, extended amygdala, and PFC [126]. These adaptations in key brain areas are integral to the three recurring stages of the addiction cycle: 1) binge/intoxication, characterized by the acute rewarding and reinforcing effects of drug consumption; 2) withdrawal/negative affect, involving unpleasant physical and emotional symptoms during abstinence; and 3) preoccupation/anticipation, marked by craving the drug in response to internal and external cues [127]. Notably, the interconnectedness across these stages may elucidate the pattern of opioid-seeking behavior, involving heavy consumption followed by periods of abstinence and, ultimately, relapse, often to alleviate negative emotionality [128] or in response to anticipatory motivation. However, it is worth noting that the exact mechanism of action underlying the molecular neurobiological events associated with the euphoric effects of opioids remains a topic of debate and controversy, with potential variations depending on the specific substance or behavior of choice.

However, the role of dopamine signaling, particularly in neurons projecting from the well-characterized VTA to the NAc, is paramount in mediating associations between drug-taking and reward, thereby reinforcing these associations each time drug consumption is repeated and strengthening the links between the drug and internal as well as external cues. Numerous clinical studies, alongside extensive exploration in animal models conducted globally, have provided a robust framework for understanding the neuronal and neurochemical effects of drugs on this dopaminergic VTA-NAc circuit [64–130]. While there has been some debate over the past two decades regarding the precise role of dopamine in pleasure states and hedonic tone [131], with suggestions that other neurotransmitters, such as endogenous opioids and cannabinoids, may play a more significant role, we, to some extent, disagree. It is challenging to conceive of the brain as being compartmentalized in a manner that operates in discrete, segregated domains, with each neurotransmitter exclusively responsible for specific behaviors rather than functioning within a broader (BRC).

illustrates the interaction of some well-known (BRC) neurotransmitter pathways. Environmental stimulation initiates the release of serotonin in the hypothalamus, which in turn, for example, via 5 HT-2 A receptors activates (green equal sign) the subsequent release of opioid peptides from opioid peptide neurons. Then, in the Substantia Nigra, the opioid peptides move to two different opioid receptors with different effects. One is through the MOR that inhibits (red hash sign) GABAA neurons (possibly via an opioid peptide like enkephalins). The second stimulates cannabinoid neurons (for example, the Anandamide and 2-arachidonoylglycerol) (green equal sign) through beta-endorphin-linked delta receptors, which inhibit GABAA neurons. When activated, cannabinoids, primarily the 2-arachidonoylglycerol neurons, can disinhibit (green hash sign) GABAA neurons indirectly by G1/0 coupled to CB1 receptor activation. The Glutamate neurons in the DRN disinhibit GABAA neurons in the Substantia Nigra indirectly through GLU M3 receptor activation (green hash sign). When disinhibited, GABAA neurons will powerfully inhibit (red hash signs) VTA glutaminergic drive via GABAB 3 receptors. At the NAc, Acetylcholine (ACH) neurons may inhibit (red hash sign) muscarinic and stimulate nicotinic (green hash) receptors. Glutamate neurons in the VTA will project to dopamine neurons through (NMDA) receptors (green equal sign) to definitively release dopamine at the NAc [132].

From our perspective, the debate regarding the precise role of dopamine in reward processing remains ongoing, although we have proposed that craving dopamine represents a distinct phenomenon from liking it. Blum et al. [133] explored the three primary explanatory frameworks—liking, learning, and wanting-to resolve the debate surrounding the causal contributions of mesolimbic dopamine circuits to reward. By imbuing reward-related stimuli with incentive salience, dopamine may facilitate: a) the hedonic effect of reward (liking), b) learned predictions about rewarding outcomes, and c) the pursuit of rewards (wanting). Blum’s group [134] finds that the incentive salience or “wanting” hypothesis of dopamine function is supported by the preponderance of evidence, although we acknowledge that the traditional notion of dopamine’s role in pleasure induction may require refinement. This is particularly relevant in the context of RDS, where neuroimaging studies have revealed alterations in the brain’s reward system elicited by addictive substances, palatable meals, and anticipated behaviors such as sex and gaming, suggesting bidirectional effects [135].

It is widely accepted that addictive substances share three standard features: voluntary self-administration, enhancement of dopaminergic synaptic activity in the NAc, and activation of the brain’s reward system to produce the sought-after “high” in drug users. Increased stress levels, dopaminergic genes polymorphisms, and other genetic variations in neurotransmitter systems are believed to collectively contribute to an individual’s vulnerability to addiction. These circuits are now recognized as functionally more complex, encoding attention, reward anticipation, disconfirmation of reward anticipation, and incentive motivation, in addition to their previously attributed role in setting the hedonic tone. Research suggests that dopamine is more closely associated with “wanting” than “liking,” as proposed by our group and others [136], who have suggested that dopamine is linked to “wanting” in obese populations, while opioids are associated with “liking.”

In line with this perspective, File et al. [137] investigated how individuals’ experiences of “wanting” and “liking” change in response to substances and behaviors, essential components of the Incentive Sensitization Theory (IST). Assessing four substances (alcohol, nicotine, cannabis, and other drugs) and ten behaviors, File et al. [137] examined the dissociation between “wanting” and “liking” based on usage frequency, intensity, and subjective severity across various activities (e.g., gambling, overeating, gaming, pornography use). Based on structural equation modeling with 749 participants, their findings indicate that “wanting” increases with the severity, frequency, and intensity of problematic use while “liking” remains relatively stable. Moreover, they observed that women exhibit higher levels of “wanting” compared to men, and both “wanting” and impulsivity positively predict problem usage and behaviors. In contrast, impulsivity and problematic behaviors negatively impact well-being, and reward deficit positively predicts problematic behaviors.

Casey et al. [138] observed that the DSM-5, while serving as a primary diagnostic tool, often compartmentalizes disorders as distinct entities despite their potential interconnectedness. They argue that the boundaries between disorders are frequently more fluid than the DSM suggests. Instead, they advocate for a different approach to studying mental illnesses, made feasible by the Research Domain Criteria (RDoC) project initiated by the US National Institute of Mental Health (NIMH) in 2013. The RDoC project delineates five “domains,” each representing a brain region whose functionality is implicated, to varying extents, in various mental disorders.

A hallmark feature of SUD, including opioid abuse and dependence, particularly in its more severe forms such as addiction, is craving—an overwhelming urge and compelling need to use a substance following a period of abstinence, whether brief or prolonged. This phase of preoccupation and anticipation in the addiction cycle involves the intricate interplay of dopaminergic pathways in the PFC, a brain region closely associated with goal-directed behaviors, self-control, and decision-making, along with the extended amygdala and basal ganglia. Despite ongoing efforts through comprehensive Genome-Wide Association Studies (GWAS) and advanced neuroimaging techniques to unravel the complexities of addiction, skeptics like Lewis [139] advocate for a “learning” model over a purely biological framework. In light of this evidence, caution should be exercised. Furthermore, dopaminergic signaling in the basal ganglia can be augmented through various mechanisms, including those induced by psychoactive drugs like opioids: direct or indirect stimulation of VTA-dopamine neurons, increased release of dopamine at terminals, inhibition of dopamine reuptake, or amplification of postsynaptic dopamine actions on NAc neurons. A common neuroplastic mechanism underlying the chronic administration of most drugs involves an increase in the ratio of AMPA receptors to NMDA receptors, facilitating heightened excitatory transmission onto VTA-dopamine neurons [140].

### Epigenetic factors of causality

According to the Harvard University Center on Developing Child’s article on epigenetics [141], environmental factors and children’s early experiences can influence their genetic expression. SUD, previously termed drug addiction, serves as a compelling example of environmental modification of gene expression. Volitional, repeated drug use impacts the chromatin landscape within the brain in a region- and cell-type-specific manner, prompting increased interest in studying epigenetics [107].

It is hypothesized that these drug-induced epigenetic changes contribute to aberrant cellular function, perpetuating the pathophysiology of psychoactive substance dependency by regulating DNA-related processes. Nestler’s group previously proposed [142] that targeting necessary drug-induced epigenetic alterations within the brain with therapeutic interventions could potentially prevent an individual’s descent into a severe drug-dependent state. Accordingly, Hamilton and Nestler [142] emphasize that understanding the neuronal signaling complexity requires consideration of the diversity of epigenetic modifications. One significant epigenetic insult in adverse environments occurs through histone post-translational modifications (PTMs), such as methylation and dopaminylation [143, 144].

Chromatin, a macromolecular complex, consists of nucleosomes formed when DNA tightly coils around histone protein octamers. Chromatin serves as an instructional scaffold that responds to environmental signals and enables the dense packing of nucleosomes within the cell nucleus. The rich lysine and arginine residues in histones are particularly noteworthy. PTMs of these and other residues on histone N-terminal tails, which protrude from the nucleosome core, alter the steric characteristics and charge distribution of chromatin, regulating DNA-involved processes. Acetylation, methylation, phosphorylation, ADP ribosylation, ubiquitylation, and sumoylation are among the PTMs that modify histone subunits, occurring on over 50 different sites [30, 145].

Histone PTMs are reversible, as elucidated by Allis et al. [143]. These modifications are dynamically deposited by “writer” enzymes, recognized by “reader” proteins that govern the physiological response, and removed by “eraser” enzymes. A novel area of interest for anti-addiction epigenetic therapies involves restoring normal function to these proteins through small molecules or nutraceutical-type complexes that aim to balance, for example, dopamine [26, 27, 60, 65, 146]. Various writers, erasers, and readers have been identified to alter expression and function in addicted humans and animal models of addiction [147–149].

In terms of epigenetic insults, newborns of probands who use illicit and licit substances during pregnancy are at risk for withdrawal, known as Neonatal Abstinence Syndrome (NAS). In the United States, the documented prevalence of NAS has risen from 4.0 per 1000 hospital births in 2010 to 7.3 per 1000 hospital births in 2017, representing an 82% increase. It is important to note that the management of NAS is multifaceted and involves a combination of nonpharmacologic and pharmacologic therapy [150].

### Epigenetics of OUD

OUD exhibits high heritability, as demonstrated by Zhang et al. [151], who identified several methylation quantitative trait loci (mQTLs) in the DRD1 and DRD2 genes in both individuals with heroin use disorder and healthy controls. Notably, specific SNP-CpG pairs in the DRD1 gene were uniquely associated with patients with heroin use disorder, indicating potential associations between dopaminergic mQTLs, DNA methylation, and gene expression in OUD traits, although further control screening is necessary for robust analysis [152].

Current evidence indicates that chronic opioid use promotes alterations in the brain’s reward circuitry, characterized by increased permissive histone acetylation levels, decreased repressive histone methylation, and changes in DNA methylation patterns and noncoding RNA expression [153]. These neuroadaptations persist at various levels, including mRNA, neuropeptide, neurotransmitter, and protein levels, and are influenced by both internal and external environmental factors such as stress responsivity, addictive mindset, and social context. Moreover, specific genetic variants, including those encoding pharmacological target proteins or mediating neuroadaptations, contribute to vulnerability at different stages of addiction [154].

Accumulating research supports the notion that recurrent opioid addiction induces alterations in DNA methylation/hydroxymethylation processes and post-translational histone modifications across multiple brain regions [155]. Genes such as the opioid receptor mu 1 (OPRM1) the delta-opioid receptor (OPRD1), the dopamine D2 receptor (DRD2), and brain-derived neurotrophic factor (BDNF) have received significant attention in this regard. While variants in these genes have been associated with modest but reproducible effects on OUD risk, recent have identified potential associations with additional genetic variants such as KCNG2, KCNC1, CNIH3, APBB2, and RGMA. However, the identified genetic associations only explain a small fraction of OUD risk [156]. Moreover, heroin use can lead to male infertility by inducing conditions such as leukocytospermia, asthenozoospermia, elevation in DNA fragmentation index (DFI) in sperm cells, and alterations in seminal RNA profiles [157].

Long-term opioid use has consistently been linked to hypermethylation of the opioid receptor mu 1 (OPRM1) promoter. To explore whether DNA methylation changes occur after just a few days of prescribed opioid use, a study categorized participants based on cumulative dosage into three groups: < 25 MME, 25 – 90 MME, and ≥ 90 MME. Using mixed-effects modeling, the study found significant positive associations between opioid dose and methylation levels at four CpGs, with nine out of ten OPRM1 promoter CpG showing higher methylation in higher dose groups compared to the lowest dose group. After adjusting for age, cellular heterogeneity, and past tobacco use, the study also revealed positive associations between promoter methylation means and cumulative MME (regression coefficient = 0.0002, one-tailed p-value = 0.02) as well as duration of opioid use (regression coefficient = 0.003, one-tailed p-value = 0.001). Additionally, a preliminary epigenome-wide association study identified a significant CpG in the promoter of the RAS-related signaling gene, RASL10A, which may serve as a predictor of opioid dosage. Furthermore, adrenergic signaling has been implicated in OUD. Zhang et al. [158] investigated the association between methylation alterations in the alpha-1-adrenergic receptor (ADRA1A) gene and OUD. They compared methylation levels of 97 CpG sites in the promoter region of the ADRA1A gene in the peripheral blood of 120 patients with heroin use disorder and 111 healthy controls. The study revealed that hypermethylation in the promoter region of the ADRA1A gene in the blood was associated with OUD, with correlations observed between methylation levels of several CpG sites and the duration of heroin/methadone use. Transcription factors TFAP2A and RUNX1 were predicted to bind to the target sequences, including the CpG sites.

In light of the OUD epidemic and rising overdose deaths, it is crucial to understand the underlying mechanisms contributing to addiction. As mentioned above Gondre-Lewis et al. [119] proposed that alterations in brain reward circuits affecting mesocorticolimbic dopamine release may underlie addictive behavior across different substances. Their research suggests that genetic or epigenetic changes in dopaminergic reward circuits contribute to opioid and other drug self-administration. For example, they found evidence supporting a greater frequency of the polymorphic DRD3 risk allele (rs6280) compared to the opioid receptor μ1 in chronic opioid users (rs1799971), particularly among African Americans. These findings imply that dopamine-related receptors may play a primary role in influencing opioid-seeking behavior among African Americans, highlighting the importance of targeting DRD3-mediated modulation of dopaminergic homeostasis in developing novel neuropharmacological therapies for OUD.

Among opioid overdose cases, Corradin et al. [159] observed global hypoacetylation and identified 388 putative enhancers consistently depleted for H3K27ac. Employing a convergence analysis strategy based on promoter-capture Hi-C, they identified five genes—ASTN2, KCNMA1, DUSP4, GABBR2, and ENOX1—that were over-burdened by alterations in their regulatory network or “plexus.” Notably, these convergent loci are enriched for OUD risk genes, heritability for generalized anxiety, and number of sexual partners.

Serotonin (5-HT) is implicated in the reward processes underlying SUD. Li et al. [160] investigated DNA methylation in the promoter region of serotonin-related susceptibility genes in 120 patients with heroin use disorder and 111 healthy controls. They found that rs6296 in the HTR1B gene was correlated with susceptibility to heroin use disorder and identified gene–gene interactions between HTR1B and HTR2A. Hypermethylation was observed in the CpG sites HTR1B_07 and HTR1B_26 and the promoter region of the HTR1B gene in patients with heroin use disorder compared to healthy controls. Notably, rs6296 correlated allele-specifically with methylation in the HTR1B gene promoter in the blood and gene expression of the HTR1B gene in the frontal cortex and hypothalamus, suggesting an association with OUD through mechanisms involving DNA methylation and HTR1B gene expression. Furthermore, fentanyl induced autism-like behaviors in both young male and female mice, downregulating Grin2b expression and GluN2B protein levels in the anterior cingulate cortex through hypermethylation of Grin2b. The MOR antagonist naloxone and overexpression of Grin2b in the anterior cingulate cortex attenuated fentanyl-induced effects. However, DAMGO injection into the anterior cingulate cortex induced autism-like behaviors. These findings suggest that fentanyl induces autism-like behaviors in young mice via an epigenetic mechanism [161]. This induction may have unwanted effects that could lead to an increase in opioid seeking behavior.

### Risk factors vs Protective factors

To elucidate the origins and pathways of substance abuse and addiction, researchers have identified factors distinguishing individuals more prone to substance abuse from those less likely to engage in such behaviors. “Risk factors” increase the likelihood of substance abuse, whereas “protective factors” decrease this probability [162]. These factors can influence children along developmental trajectories or pathways, capturing risks at various stages of life. For example, early risks like uncontrolled aggressive behavior in young children can escalate when they enter school, potentially leading to peer rejection, teacher punishment, and academic failure. Left unaddressed, these risks may culminate in behaviors like school truancy and association with substance-abusing peers, putting children at immediate risk of substance abuse. By targeting risk factors, evidence-based prevention programs can intervene early in a child’s development to bolster protective factors and mitigate risks long before problematic behaviors emerge.

Adolescent substance abuse and addiction risk factors predominantly stem from external influences; however, certain individual factors also play significant roles in the likelihood of developing a SUD. Among these individual risk factors, attention deficit hyperactivity disorder (ADHD) and depression are frequently highlighted in the literature [74, 163]. Additionally, individuals diagnosed with post-traumatic stress disorder (PTSD) or other psychiatric illnesses face an elevated risk of adolescent substance abuse. While individual factors such as sexual orientation and ethnicity have been explored as potential contributors, findings in the literature are often inconclusive [164].

Early interventions targeting risk factors, such as aggressive behavior and lack of self-control, have a more profound impact than later interventions in redirecting a child’s life trajectory away from problems and toward positive behaviors. While risk and protective factors can influence all individuals, their effects may vary based on age, gender, race, culture, environment, and significantly, genetic predispositions [165, 166].

While identifying risk factors is paramount, emerging evidence suggests protective mechanisms may be heritable. For instance, recurrent cocaine use induces alterations in histone acetylation and methylation on Lys residues and DNA within the NAc, albeit not specifically with opioids. Nestler’s group investigated histone Arg (R) methylation in models of reward processing. According to Damez-Werno et al. [167], the protein-R-methyltransferase-6 (PRMT6) histone mark and asymmetric dimethylation of R2 on histone H3 (H3R2me2a) decreased in the NAc of rodents and cocaine-dependent humans during self-administration procedures. Overexpression of PRMT6 in D2-expressing medium spiny neurons (D2-MSNs) increased cocaine craving, whereas D1-MSNs overexpression of PRMT6 suppressed cocaine seeking. The theory suggests that dopaminylation (H3R2me2a binding) occurs in psychostimulant use disorder (PSU), and the binding inhibitor Srcin1 mitigates psychostimulant-seeking behavior by normalizing NAc dopamine production, akin to the significant DRD2 A2 allelic polymorphism.

Several studies have linked severe cocaine dependence to the DRD2 Taq A1 allele, characterized by fewer D2 receptors. Acute cocaine elevates dopamine levels in NAc synapses, inducing histone H3 glutamine five dopaminylation (H3Q5dop) and subsequently suppressing D2 expression [168]. Chronic cocaine use exacerbates inhibition, a phenomenon persisting after cocaine withdrawal. Additionally, they discovered that during cocaine withdrawal, the Src kinase signaling inhibitor 1 (Srcin1 or p140CAP) decreased H3R2me2a binding. Consequently, the inhibited dopaminylation led to a “homeostatic brake.” Similar to the DRD2 Taq A2 allele, a well-known genetic mechanism protecting against SUD, the reduction in Src signaling in NAc D2-MSNs normalizes the production of NAc dopamine. This lowers cocaine reward and motivation to self-administer cocaine, indicating that Srcin1 may be a crucial therapeutic target. Additionally, there is evidence that individuals with the DRD2 A2 allele are protected from developing widespread RDS traits. In a study involving 66 brains of individuals with and without AUD, Noble et al. [169] investigated the allelic relationship of the human D2 dopamine receptor gene with the binding properties of the D2 dopamine receptor. In a blind experiment, a clone (lambda hD2G1) of the human D2 dopamine receptor gene was probed with a 1.5 kilobase (kb) digest of DNA from the cerebral cortex after treatment with the restriction endonuclease TAq. The caudate nuclei of these brains were used to assess the binding properties (Kd [binding affinity] and Bmax [number of binding sites]) of the D2 dopamine receptor using tritiated spiperone as the ligand. Alcoholic subjects had a considerably lower adjusted Kd than non-alcoholic subjects. The Bmax was much lower in individuals with the A1 allele, strongly associated with alcoholism, than in individuals with the A2 allele (protective). Additionally, participants with the A2/A2 allele, A1/A2, and A1/A1 alleles all had progressively lower Bmax values, with A2/A2 subjects having the highest mean values and A1/A1 subjects having the lowest. Many models of addiction causation provide a framework inclusive of biological, social, medical, and psychological factors to understand the complex world of addictions within the multidisciplinary medical, behavioral health, and addictions milieu. For many people who struggle with addiction, identifying a reason or cause is often an essential first step in moving toward treatment and recovery [170].

### The etiological root of OUD and other reward-dysregulated disorders

Of the various models providing a therapeutic framework for addiction, the medical model recognizes addiction as an illness or disease akin to any other physiological ailment. It emphasizes presenting symptoms, test results, and treatment to attain identifiable outcomes. Within this paradigm, addiction is no longer perceived as a matter of choice, similar to other illnesses such as cancer, diabetes, or seizures. Instead, all addictions share a biological dimension regardless of the drug of choice [171].

The strength of the medical model lies in explaining the question “Why me,” akin to how we inquire about other diseases. It acknowledges addiction as necessitating treatment like any other illness. For many grappling with the complexities of addiction, this model instills hope that appropriate treatment can alleviate this disease. While the medical model alone may confine addiction to a medical issue, its efficacy is enhanced when integrated with other addiction models, accommodating additional influences such as neurobiological factors, genetic antecedents, epigenetic insults, personal experiences, support systems, familial environments, and social contexts into treatment approaches.

The medical model diverges from the social milieu of a historically punitive blaming culture that misconstrues individuals with addiction. Social constructs breed a dismissive mindset, attributing self-blame to the individual for their addiction initiation, which is a prevalent misconception.

Irrespective of the preferred drug, the hallmark of addiction is the recurrent engagement in detrimental behaviors despite ongoing adverse consequences. Addiction typically encompasses the following four conditions:

- Preoccupation and involvement in drug use.
- Loss of control over drug use.
- Inability to sustain sobriety or recovery.
- Intense craving for the drug of choice.

Addiction is considered a ‘family disease,’ impacting all nuclear family members. Relationships within the family unit often become strained, if not severely compromised, during the active drug use of a family member. Recovery efforts are frequently hampered by familial discord and a lack of familial involvement and support. Familial support systems are indispensable for successful intervention, treatment, and recovery, serving as fundamental pillars of the recovery foundation [172].

Acknowledging the role of the social environment is crucial to understanding the genesis of addiction. Social environments exert significant influence both in addiction development and in endeavors to establish and sustain recovery. Recovery is jeopardized if an individual returns to an environment that perpetuates drug use as a prerequisite for social inclusion. These factors are pertinent concerns for individuals grappling with addiction, particularly those embarking on the journey from trauma to healing [173].

Addiction stands apart from other illnesses in its unique propensity to evoke intrusive thoughts of self-blame and self-loathing. These thoughts perpetuate a pervasive negative social construct, echoing the mantra: ‘It was your choice to use drugs. It’s your fault you have an addiction.’ Regrettably, this message undermines all efforts toward treatment and recovery. Interestingly, there’s evidence suggesting that changing the name of a disorder can reduce the stigma and guilt associated with it; for instance, using the term “Isoquinolism” instead of “alcoholism” has shown promise [96]. The escalating crisis of opioid misuse in many countries raises a crucial question about the role of trauma, such as injuries, in driving opioid-seeking behavior [174].

Self-loathing perpetuates a debilitating cycle of negative self-harm that, if unchecked, gains momentum and impairs an individual’s ability to (1) acknowledge addiction, (2) seek treatment, and (3) establish and maintain recovery. Understanding the multifaceted layers of addiction is best achieved within a broader bio-psycho-social-spiritual framework [175]. Within this framework, it’s essential to meticulously evaluate the bio-psycho-social-spiritual aspects of the human condition to discern their contribution to addiction development. Furthermore, within this holistic approach, acknowledging the significance of all facets of an individual’s life is paramount in comprehending addiction and the individual’s preferred drug choice. Effective treatment and the commencement of the healing process necessitate embracing a holistic perspective to reinforce and support the recovery journey. Notably, on a neurological level, the mirror neuron system, the default mode network (DMN), reward deficits (RDS) (hypodopaminergia), and spirituality are intricately interconnected. One potential therapeutic goal, perhaps through meditation, as previously mentioned, could involve releasing dopamine and enhancing the neuro-spiritual connectome through the DMN [176].

### Trauma and psychoactive seeking behavior

The escalating crisis of opioid misuse in many countries prompts an essential inquiry into the role of trauma, such as injuries, in driving opioid-seeking behavior [177]. Compelling evidence underscores the association between Interpersonal Trauma (IPT) and drug abuse [178]. Williams et al. [178] conducted a cross-sectional online survey involving 235 individuals with a self-reported history of IPT, including intimate partner violence, sexual assault, and adverse childhood experiences. Multinomial regression models were utilized to examine the co-occurrence of IPT and its interactions with opioid use and misuse indicators. The study revealed a link between IPT and opioid abuse, with the relationship between sexual assault and opiate abuse being particularly complex due to exposure to various IPTs. Among men, opioid abuse was associated with intimate partner violence, whereas among women, adverse childhood experiences and combined sexual assault and intimate partner violence were linked to increased opioid usage.

A study conducted by von Oelreich et al. [179] delved into trauma as a potential factor associated with OUD. Using linkage to Swedish health registers, opioid consumption was evaluated both before and after trauma incidents. Among injured patients, logistic regression was employed to explore factors linked with chronic opioid use. Specifically, the analysis involved 13,309 injured patients and 70,621 controls. The findings revealed that exposure to trauma was independently associated with regular opioid use (odds ratio 3.28, 95 percent CI, 3.02 to 3.55). This usage was correlated with factors such as age, low level of education, somatic co-morbidity, psychiatric co-morbidity, pre-trauma opioid use, and severity of injury. Consequently, the authors postulate that traumatic injury correlates with chronic opioid use and poses an elevated risk of mortality within 6 – 18 months post-trauma.

The intensity of PTSD symptoms may also be linked to opioid abuse and dependency in trauma-exposed individuals with chronic pain, potentially influenced by anxiety sensitivity. However, the precise nature of this relationship remains unclear. Zvolensky et al. [180] conducted a study involving 294 adults who had experienced trauma and had chronic pain. Their findings provided initial evidence suggesting that the severity of posttraumatic stress symptoms correlates with anxiety sensitivity, which, in turn, is associated with increased opioid abuse and dependency among trauma-exposed individuals with chronic pain. Notably, regarding opiate abuse and dependence, statistically significant indirect effects of posttraumatic stress symptom intensity via anxiety sensitivity were observed.

In the last three decades, the rates of opioid prescriptions have quadrupled, paralleled by a concerning rise in hospital admissions due to overdoses. Harmon et al. [181] conducted an evaluation involving one thousand six hundred forty-nine patients (age ≥18 years) with multiple trauma admissions, among whom seven hundred nine had opioid toxicology screen (TS) data for both admissions. The findings suggest that a prior history of opioid use emerges as the most robust predictor of recurrent use among recidivists, potentially attributable to genetic antecedents predisposing to heightened opioid risk.

Prior studies have established a link between the misuse and use of prescription opioids [182]. A comprehensive data analysis in March 2020 utilized Waves IV (2008) and V (2016–2018) of the National Longitudinal Study of Adolescent to adult health (N = 10,685). It revealed that women (OR = 1.68, 95% CI = 1.19, 2.39 for use; OR = 1.18, 95% CI = 0.95, 1.55 for misuse) and men (OR = 2.37, 95% CI = 1.37, 4.12 for use; OR = 1.71, 95% CI = 1.06, 2.75 for misuse) were more likely to use and misuse prescription opioids in the aftermath of sexual assault. Diagnoses of depression and anxiety among women (p = 0.0420, p = 0.0450) and prescription opioid abuse (p = 0.0210) mediated the link with prescription opioid use. In men, diagnoses of depression (p = 0.0390) and anxiety (p = 0.038) mediated the connection between prescription opioid misuse and use. Secondary prevention initiatives providing survivors of sexual assault with evidence-based, trauma-informed behavioral health care may mitigate prescription opioid abuse and misuse as coping mechanisms [182]. Notably, opioids modulate mesolimbic dopaminergic pathways in the VTA through the activation of MOR on secondary interneurons, leading to increased dopamine release [183].

The Colorado Center for Antisocial Drug Dependence (CADD) has extensively investigated the genetic underpinnings of teenage antisocial drug use using various research designs and approaches. Through gene-based permutation tests, OPRM1 rs495491 emerged significantly as a credible candidate associated with antisocial drug dependence (p-value < 0.006). Intriguingly, a link between opiates and aggression, particularly self-directed hostility, has been established. Moreover, metenkephalin levels have been correlated with self-harming behavior, although opiate antagonists often mitigate it. Self-harming activities in borderline personality disorder patients have been associated with attenuated cerebral spinal fluid (CSF) endogenous opioid concentrations [184].

It is widely acknowledged that lower opioid levels may correlate with heightened rejection sensitivity, abandonment/separation anxiety, and aggression. Importantly, when opiates are released in the context of self-destructive behavior, reduced presynaptic opiate activity may upregulate postsynaptic-opioid receptors, potentially leading to significant pain alleviation. According to additional research by Cimino et al. [185], mothers and children with the G-allele (G/G + A/G genotypes) were more likely to exhibit an insecure attachment style. G-allele children outperformed homozygous A/A children on the clinical sample’s withdrawal and conduct problems subscales. Additionally, compared to mothers with the A/A allele, mothers with the G-allele displayed heightened interpersonal sensitivity, hostility, depression, paranoid ideation, and hostility while providing less care.

People with the G-allele typically endure higher social pain [183, 186] and experience heightened emotional dysregulation and brain activation due to social rejection. Unlike A/A homozygotes, individuals with the G-allele also demonstrate elevated levels of RSD, exhibit behavioral retraction to angry faces, and display high levels of fearful attachment regardless of the quality of their early maternal care [187, 188]. This underscores how the A118G-genotype modifies the effects of early maternal care on adult attachment style.

Regarding trauma and OUD, while the referenced literature offers valuable insights into potential therapeutic targets and prevention strategies, unconventional perspectives can also yield heuristic value and critical clinical insights. Gabor Mate [189], for instance, conceptualizes addiction within the framework of the Buddhist mandala wheel of life, where the suffering of addiction resides in the Realm of the Hungry Ghosts. Individuals trapped in this realm perpetually struggle to satiate their cravings for their substance of choice and alleviate the pain and suffering they constantly endure despite facing adverse consequences.

Bessel van der Kolk [190] emphasizes the importance of closely examining the drivers behind substance use, particularly among traumatized individuals who often grapple with feelings of restlessness, chest tightness, and profound agitation. In essence, traumatized individuals and addicts alike seek relief from the physical and emotional anguish they endure, using drugs out of desperation. Trauma fundamentally alters the landscape of causality, treatment, and recovery in addiction. The interplay between trauma and addiction is profound and inseparable; thus, successful treatment and recovery necessitate a concerted focus on both domains. Failure to adopt an integrated approach undermines the efficacy of interventions, as each dynamic has the potential to sabotage the other [191].

The aftermath of personal traumatic events reshapes every facet of a traumatized individual’s life, often precipitating addiction. Trauma survivors contend with a myriad of psychological sequelae, including panic attacks, relentless anxiety, debilitating self-shame, and chronic night terrors or nightmares. While it took time for the field of addiction to acknowledge trauma’s role, robust correlations between trauma and addiction have since been established [192]. Consequently, addressing one disorder without attending to the other is ill-advised.

Addiction often serves as a form of self-medication aimed at numbing the overwhelming symptoms of trauma. However, addiction rarely exists in isolation; it is intrinsically linked to the precipitating event or cause, manifesting as signs and symptoms that mirror the trauma’s impact across the bio-psycho-social-spiritual spectrum [172]. Failure to recognize the profound influence of trauma on individuals grappling with addiction perpetuates the cycle of substance use [193].

In 1989, the VA’s National Center for PTSD officially recognized PTSD as a diagnosis for veterans grappling with trauma [194]. This recognition prompted the development of specialized mental health services within the VA healthcare system aimed at diagnosing and treating PTSD among Vietnam veterans struggling with addiction. This initiative shed light on the complex interplay between trauma and addiction, challenging prevailing notions about the role of substance use in coping with trauma.

Trauma, once entrenched in an individual grappling with addiction, becomes indelibly intertwined with their lived experience. Addiction often serves as the initial attempt to numb the emotional and spiritual turmoil stemming from personal traumatic events. However, trauma’s pervasive effects can be as destructive as addiction itself, exacerbating vulnerability and impeding recovery efforts [194].

Virginia Weeks [195, 196], following in the footsteps of her aunt Janet G. Travell, whose groundbreaking work on Myofascial Pain Syndrome revolutionized pain management, underscores the imperative of addressing the underlying pain fueling addiction. As Peter Levine [197] aptly observes, while humans seldom succumb to the physical toll of trauma, unresolved trauma exacts a profound psychological and emotional toll akin to a living death. Addiction, therefore, emerges as a response to the unaddressed anguish stemming from personal traumatic events. Addiction, often precipitated by personal trauma, serves a specific purpose: to alleviate the overwhelming internal pain associated with trauma that individuals may be unaware of, unwilling to acknowledge, or unable to confront. In this context, the choice of substance assumes paramount importance. The poignant words of the Irish poet and philosopher John O’Donohue encapsulate the essence of the profound suffering experienced by those navigating the aftermath of personal trauma: “One of the deepest longings of the human soul is to be seen.”

### Sleep, mental health, and chronic drug use

The relationship between sleep problems and drug use/abuse must be contextualized within the framework of psychiatric disorders. There appears to be a tripartite connection between sleep, mental health, and SUD, wherein chronic sleep deprivation—stemming from either organic sleep disorders like sleep breathing disorder, periodic leg movement in sleep, sleep bruxism, and comorbid insomnia, or psychological and behavioral factors contributing to sleep disturbances—exacerbates psychological stress, thereby increasing the risk of SUD. Opioids, for instance, are implicated in the development of central sleep apnea (CSA) and ataxic breathing. Recent findings suggest that adaptive servo-ventilation may offer an effective treatment approach for CSA associated with opioid use [198].

This association becomes particularly noteworthy in the presence of SUD comorbidities. Notably, young adolescents are reported to misuse a range of substances, including opiates, benzodiazepines, alcohol, neuroleptics, and antipsychotics [199]. Many of these substances heighten the density of rapid eye movement (REM) sleep, initially leading individuals to perceive their efficacy in improving sleep quality. However, chronic opioid misuse may increase REM density, consequently augmenting emotional memory processing and exacerbating cravings for continued drug use [200]. Conversely, individuals experiencing sleep deprivation and resultant excessive daytime sleepiness may resort to overusing stimulants to alleviate hypersomnolence symptoms. Chronic stimulant use, including amphetamines, poses another significant challenge in SUD management [201–203].

To mitigate cravings, many addiction medicine practitioners prescribe selective serotonin reuptake inhibitors (SSRIs) or serotonin/norepinephrine reuptake inhibitors (SNRIs). These medications are believed to reduce craving partially by decreasing REM density, potentially dampening emotional memory processing during REM sleep and subsequently mitigating cravings [203]. The SUD epidemic appears to be partly intertwined with sleep-related issues, underscoring the importance of incorporating sleep assessments into the primary screening of young adolescents for their substance use risk profile. Utilizing questionnaires such as the Pittsburgh Insomnia Rating Scale (PIRS), Pittsburgh Sleep Quality Index (PSQI), and Epworth Sleepiness Scale (ESS) can aid in identifying chronic insomnia, assessing sleep quality, and gauging daytime sleepiness among adolescents. Should initial screening results indicate significant concerns, objective evaluations like overnight polysomnography (PSG), multiple sleep latency test (MSLT), and maintenance of wakefulness test (MWT) become warranted [204].

Insomnia and sleep phase disorders are increasingly recognized as prevalent issues among young adolescents. Longitudinal studies involving adults have demonstrated that insomnia can significantly predict the onset of substance abuse, highlighting the importance of addressing chronic sleep disturbances in this demographic [205]. When considering treatment approaches, caution is warranted regarding the use of hypnotic medications, especially for young patients with chronic insomnia or those with medical/SUD comorbidities. In such cases, cognitive behavioral therapies and potentially transcranial magnetic stimulation for insomnia (CBT-I) are preferred over pharmacotherapy. Melatonin, a popular choice, exerts regulatory effects on the disrupted sleep-wake cycle rather than exerting hypnotic effects. However, caution should be exercised when prescribing melatonin for sleep, particularly in individuals at high risk for alcoholism [206]. A newer class of medications known as dual orexin receptor antagonists (DORA), primarily used for primary insomnia, holds promise due to their lower abuse potential. These medications, exemplified by the FDA-approved suvorexant, offer a potentially safer option for treating insomnia, particularly in individuals with SUD or those at high risk for substance abuse [207].

Sleep plays a crucial role in preventing disorders associated with substance use, as outlined in the DSM-V. Certain sleep problems have been linked to specific dopaminergic gene polymorphisms, underscoring the intricate relationship between sleep and neurotransmission. Ensuring “normal dopamine homeostasis” is essential for individuals in recovery from addiction, as sleep is implicated in the metabolic clearance of neurotoxins from the brain. Continued research into the science of sleep holds promise for reducing sleep deprivation, particularly in the context of SUD like OUD [208]. Psychoactive drug abuse can significantly impact sleep patterns, with heroin addicts commonly reporting sleep disturbances, including insomnia, as triggers for relapse [208]. Methadone maintenance therapy has been associated with Sleep Disordered Breathing (SDB) [209].

Cytokines, which regulate sleep and central nervous system (CNS) functions, including opioid systems and food consumption, play a role in sleep regulation [210]. Moreover, while the dorsal horn of the spinal cord and the medulla are recognized as primary ascending pathways for pain, emerging evidence suggests that other neurological structures, particularly the mesolimbic system of the brain (a reward center), may also modulate pain perception, similar to sleep-related issues [210]. This highlights the interconnectedness of sleep, pain, and SUD, warranting further investigation into their complex interactions.

### Pain mechanisms

A multitude of genes and their associated polymorphisms have been implicated in affecting pain tolerance and sensitivity, potentially offering specific therapeutic targets for pain management. By conducting pharmacogenetic testing on certain candidate genes, such as mu receptors and PENK, tailored pharmacogenomic treatments may be developed to enhance clinical outcomes [211]. Numerous candidate genes, including those for catechol-O-methyltransferase, melanocortin-1 receptor, guanosine triphosphate cyclohydrolase, and MOR, have been extensively investigated for their associations with pain sensitivity and the requirement for analgesics in both acute and chronic pain states [212].

The efficacy of drugs like codeine, tramadol, tricyclic antidepressants, and nonsteroidal anti-inflammatory drugs is influenced by polymorphisms in cytochrome P450 enzymes. Genetic variations in drug-metabolizing enzymes significantly impact pharmacological responses and represent important targets for ongoing research to elucidate connections between individuals’ genetic profiles and treatment responses [213]. In a study examining morphine sensitivity and tolerance in various mouse strains, including BALB/cBy, C57BL/6By, their reciprocal F1 hybrids, and seven recombinant inbred strains, it was found that morphine sensitivity and tolerance are genotype-dependent, with inheritance patterns showing partial or complete dominance [214].

For opioid dependence treatment, methadone or other ORT are commonly used, but dosages can vary widely. Low program retention rates may be attributed in part to suboptimal dosing, leading to withdrawal symptoms and increased heroin usage. The ABCB1 gene, encoding a P-glycoprotein transporter controlling CNS exposure, is involved in methadone metabolism. Individuals harboring specific haplotypes of the ABCB1 gene required significantly different methadone doses, suggesting potential clinical utility for individualized dosing based on genetic variation [215].

Studies investigating polymorphisms in the MOR gene, which encodes the receptor targeted by various opioids, have significantly advanced our understanding of the genetic factors contributing to cocaine and opiate addiction. Other genes implicated in addiction include those involved in the endogenous opioid and monoaminergic systems, such as dopamine-hydroxylase, and genes encoding dopamine, serotonin, glutamine, and norepinephrine transporters [216, 217]. Zunhammer et al. [213] conducted a linear regression study revealing a positive correlation between individual pain sensitivity and levels of the excitatory neurotransmitter glutamate and its precursor glutamine in different pain-related brain areas, contrasting with GABA [218].

Genetically induced cytochrome P450 (CYP) 2D6 inactivity affects the efficacy of opioids such as codeine, tramadol, and methadone. Moreover, mutations in the MOR gene, particularly the A118G single nucleotide polymorphism, have been associated with altered opioid potency and analgesic effects [219]. Genetic factors can also influence drug interactions, impacting opioid pharmacology. For instance, paroxetine can alter plasma concentrations of (R)-methadone in individuals with specific metabolic profiles [220]. Codeine, for example, should not be administered to individuals with poor metabolization of debrisoquine or sparteine due to genetically predisposed medication interactions. While pharmacogenetics can provide some guidance for customized opioid therapy, its utility may be limited in explaining variability in opioid dose requirements. Mutations affecting opioid receptors and pain processing may influence both analgesic efficacy and adverse effects [221].

Many genes associated with opioid addiction and pain sensitivity have been identified, including the MOR, metabotropic glutamate receptors, dopamine receptors, and various neurotransmitter transporters. The role of these genes in pain mechanisms and healing processes underscores their importance in understanding addiction and pain management [210]. For a comprehensive overview, refer to tables 1 and table 2.

**Figure 5:**
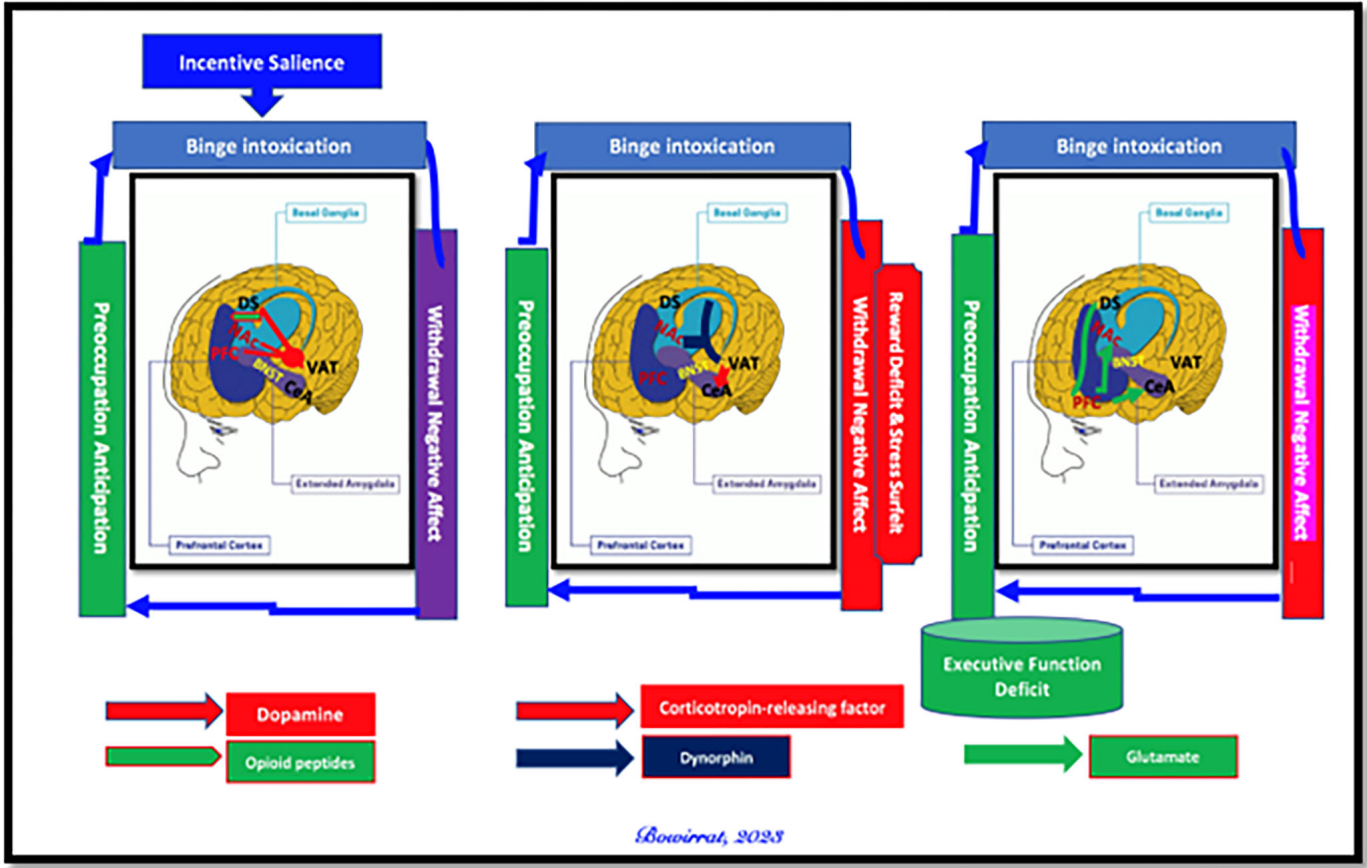
The three stages of addictive mechanisms: **Binge/Intoxication**, the stage at which a person imbibes an intoxicating psychoactive substance and experiences its rewarding or pleasurable effects, involves basal ganglia structures; **Withdrawal/Negative Affect**, the stage at which a person experiences a negative emotional state in the absence of the psychoactive substance, involves many stress hormone responses and the extended amygdala and locus coeruleus; and **Preoccupation/Anticipation**, the stage at which one seeks psychoactive agents again after a period of abstinence, involving interactions of the prefrontal cortex, the extended amygdala, and the basal ganglia. Not shown is the neurotransmitter norepinephrine, which is also activated in the extended amygdala during withdrawal. PFC - prefrontal cortex, DS - dorsal striatum, NAc - BNST - bed nucleus of the stria terminalis, CeA - central nucleus of the amygdala, VTA - ventral tegmental area. (Modified from U.S. Department of Health and Human Services (HHS), Office of the Surgeon General, Facing Addiction in America: The Surgeon General’s Report on Alcohol, Drugs, and Health. Washington, DC: HHS, 2016).

The further coupling of the identified genes, as reported in this research, and other genes related to polymorphisms would enable more pharmacologically active substance-based pharmacogenomic mapping, according to our hypothesis based on the data reviewed here. The combination will offer a framework for developing novel DNA-targeted regions, linking bioactive compounds to potential painkilling and anti-craving effects. In essence, successful individualized medical treatments for people with abnormal inborn pain sensitivity will depend on integrating known reward genes with additional physiologically based endogenous opioid receptors and/or other signaling substrates (Figure 5).

In summary, understanding the genetic factors influencing pain sensitivity and pharmacological responses to analgesics, as well as individualizing opioid replacement therapy dosing based on genetic profiles, holds promise for improving pain management and treatment outcomes in patients with pain and opioid dependence.

### Treatment

Unlike other chronic disorders, RDS or addictive behaviors have three components that must be addressed. They are physiological, psychological, and spiritual. It is important to realize that our concern related to many existing treatments for OUD may need re-thinking. One obvious issue as espoused herein is the prescribing of opioids to treat opioids. While we are cognizant of the significant reduction in immediate harm, instead of short term-administration of for example buprenorphine, many clinicians are prescribing buprenorphine for life.

### Physiological

We propose a “reward deficiency solution system” that integrates early genetic risk diagnosis, medical monitoring, and nutrigenomic DAM to address the significant global issue hindering our youth from leading productive and fulfilling lives daily. The neuroscience community is conducting excellent research employing cutting-edge molecular-genetic technologies and neuroimaging to enhance our understanding of the complex functions of brain reward circuits, which play a significant role in addiction symptomatology. Despite this progress, there is still debate over how to clinically manipulate neurotransmitters such as dopamine to treat and prevent different forms of addictive diseases, even though it is widely acknowledged that dopamine is a significant neurotransmitter implicated in behavioral and substance addictions.

Biphasic therapy, involving short-term blockage followed by long-term dopaminergic upregulation, might be a promising approach, albeit challenging in practice. For instance, the treatment of cocaine abuse with a potent D2 agonist, Bromocriptine, was initially considered by Gold’s group [232] a viable option but unexpectedly resulted in chronic prescribed Bromocriptine-induced downregulation of D2 receptors, increasing cocaine craving. Moreover, several studies have explored the use of pro-dopamine regulators to induce “dopamine homeostasis” in clinical trials.

Numerous studies have demonstrated the efficacy of buprenorphine alone or combined with naloxone (Suboxone^®^) in successfully treating opioid dependency. However, caution is advised when using these medications over an extended period due to concerns about severe withdrawal, even with dosage reduction, as well as long-term side effects such as flat affect, diversion, and suicidal thoughts observed in chronic Suboxone^®^ users. To improve treatment outcomes, it would be prudent to consider genetic testing to identify reward circuitry gene variations, particularly those related to dopaminergic pathways and opioid receptors. Understanding the connection between reward circuitry participation in buprenorphine effects and related genotypes could offer a novel paradigm to enhance a patient’s clinical experience and advantages during opiate replacement therapy.

There is limited clinical research examining the efficacy of NAD and enkephalinase infusions in treating SUD. In a study by Blum et al. [233] addicted poly-drug users resistant to conventional treatment showed substantial improvements in desire scores, anxiety, and depression following NAD injections. Linear trend analysis revealed a significant decrease in cravings, anxiety, and sadness, with no evidence of relapse in urine samples midway through the research. These findings suggest the potential effectiveness of NAD infusions in treating SUD, warranting further investigation through larger randomized, double-blinded, placebo-controlled studies. The pilot trial provides valuable early insights into the efficiency of NAD infusions in treating SUD, particularly for patients with high genetic risk identified by the GARS test and Neonatal Abstinent Syndrome (NAS).

The utilization of psychological or spiritual interventions for NAS toddlers is unlikely to yield significant effects; however, moderate neurochemical modifications using non-pharmacological methods may offer valuable heuristic benefits. For instance, by modifying KB220 variations (precursor-amino-acid-enkephalinase inhibition-therapy), GARS testing can facilitate a genuinely personalized therapeutic approach to treating OUD without OST. The formulation of enkephalinase inhibitors, enkephalin, and dopamine-releasing neuro-nutrients described in this annotated bibliography aims to induce dopamine homeostasis to detoxify and treat individuals genetically predisposed to developing RDS, as indicated by GARS test findings. The GARS test evaluates the presence of reward genes and risk alleles based on neurogenetic and epigenetic data, offering insights into the stratification of OUD-related risks before initiating opioid analgesic therapy and predicting the risk of relapse among individuals in recovery [234].

Long-term maintenance agonist medications for OUD, such as methadone and buprenorphine, may potentially induce RDS or exacerbate existing RDS that has not been previously detected. Clinical trials have demonstrated the efficacy of methadone maintenance and buprenorphine maintenance in retaining patients in drug misuse treatment and reducing the use of illegal opioids. However, caution is warranted regarding the long-term benefits or potential toxicity associated with Subutex or Suboxone. Despite its limited opioid agonist effect, chronic opiate receptor blockade, as elucidated by Blum’s group, may disrupt dopaminergic activation, leading to anti-reward and relapse potential [235].

While direct comparisons are currently unavailable, scientific literature on buprenorphine toxicity exists. In light of these considerations, it is essential to acknowledge the limitations of current approaches until more effective solutions are discovered [236]. The GARS test also assesses the potential benefits of drug-assisted treatment with dopamine augmentation, offering a promising avenue for restoring dopamine homeostasis through anti-reward allostatic neuroadaptations. This RDS/anti-addiction technique holds significant potential for alleviating the burden of addictive disorders on individuals, their families, and society as a whole.

### Neuroimaging and OUD

The role of neuroimaging as an evidence-based novel modality in patients with OUD appears to be indispensable. However, the potential of this imaging modality relevant to OUD has been partially evaluated, necessitating further studies to comprehend the underpinnings of the effects of opioids on brain function pre- and post-use and misuse. fMRI is the most widely used technique to study the human brain on a system level, assessing brain function indirectly through safe energy. Over the last two decades, fMRI has been extensively utilized to determine how different brain regions dynamically interact to influence various complex cognitive processes associated with drug addiction [237].

Numerous networks, including the PFC and subcortical limbic neurocircuitry, are implicated in reward and incentive-salience encoding, as demonstrated by both resting-state and task-based fMRI studies. The longstanding global issues of opioid dependence, including illicit heroin and prescription painkillers, as well as the treatment of OUD, persist despite intensive scientific investigation and public health care efforts. One contributing factor to the ongoing use of heroin worldwide is its highly addictive nature, and brain imaging studies over the past two decades have significantly contributed to understanding its addictive properties through biological processes, particularly those involving brain structure and function.

Moreover, traditional clinical neuropsychology studies also partially account for the treatment-refractory nature of drug abuse. Future studies on this topic aim not only to enhance existing therapeutic mechanisms based on known brain functions but also to advance pathological insight into OUD and pave the way for individual patient assignment to specific treatments based on clinically relevant neuro markers. Longitudinal studies measuring neural responses throughout therapy are crucial for the differential diagnosis of brain-based mechanisms of cognitive disorders in the context of OUD.

Current available functional brain mapping techniques for OUD assessments include fMRI and PET studies. Despite limitations, PET is recognized as a novel modality for quantitatively measuring opioid neural receptors in the CNS. Additionally, functional MRI has emerged over the last decade as a non-radiational approach for identifying brain networks in novel therapeutic drug tracking. Further research in this direction holds promise for advancing our understanding of OUD and improving therapeutic interventions.

### Neuromodulation in addiction treatment and relapse prevention

Despite significant strides in addiction treatment approaches, including counseling, group therapy, and medication, relapse rates remain alarmingly high. Bailey et al. [238] reported that 90% of patients undergoing inpatient opioid detoxification relapsed within a year of therapy, potentially due to persistent abnormal neurological alterations affecting motivation and reward prediction. Conventional treatments may only partially address maladaptive synaptic connection patterns, leaving some aberrant pathways unaffected. Addiction patients may prioritize emotional impulses over reason during times of stress or anxiety, triggering their fight-or-flight response and potentially reactivating abnormal neural pathways linked to relief sensations [239].

While researchers have yet to discover a “magic bullet” treatment for addiction, addressing fundamental neurobiological issues impacting reward anticipation, motivation, and adaptive decision-making may offer an efficient strategy for addiction treatment and relapse prevention. Future research may focus on gene editing to target DNA antecedent-induced neurotransmitter dysregulation [240–243].

Neurofeedback, a therapy involving real-time display of patients’ brain wave activity via a brain-computer interface, offers a promising avenue for addiction treatment. By creating a “neural mirror” that allows the brain to observe its activity, neurofeedback can potentially propel the brain into new configurations amenable to reinforcement and consolidation. Addiction patients who completed neurofeedback training reported dysphoria after substance use, indicating a potential change in how the brain responds to substance use [243–247].

Astrocytes, primary locations for glycogen granule storage in the CNS, have been implicated in neurofeedback’s mechanism. Neurofeedback may trigger astrocytic processes, providing essential energy substrates to neurons, thus improving self-regulation and synaptic connection pruning. Moreover, neurofeedback has shown significant improvements in executive functioning and working memory, which are critical issues among children [26, 129, 248]. Enhanced executive and regulatory abilities associated with neurofeedback training may serve as a buffer against relapse by addressing decreased functional connectivity and poor executive functions like working memory [145, 249–256]. Additionally, neurofeedback training may promote relaxation and better-perceived stress control, further contributing to relapse prevention [257–259].

**Figure 6:**
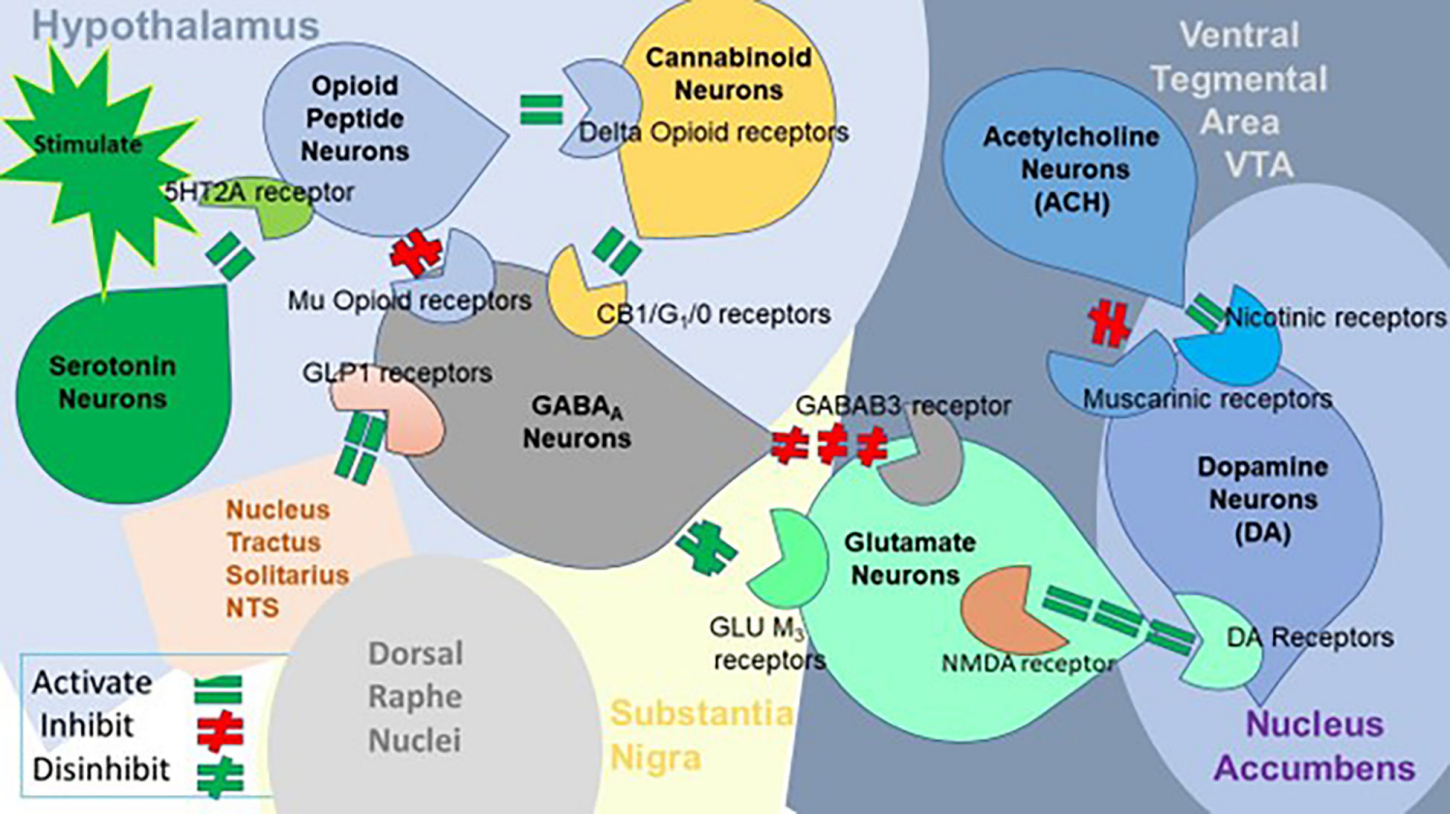
The mesolimbic Brain Reward Cascade.

**Figure 7:**
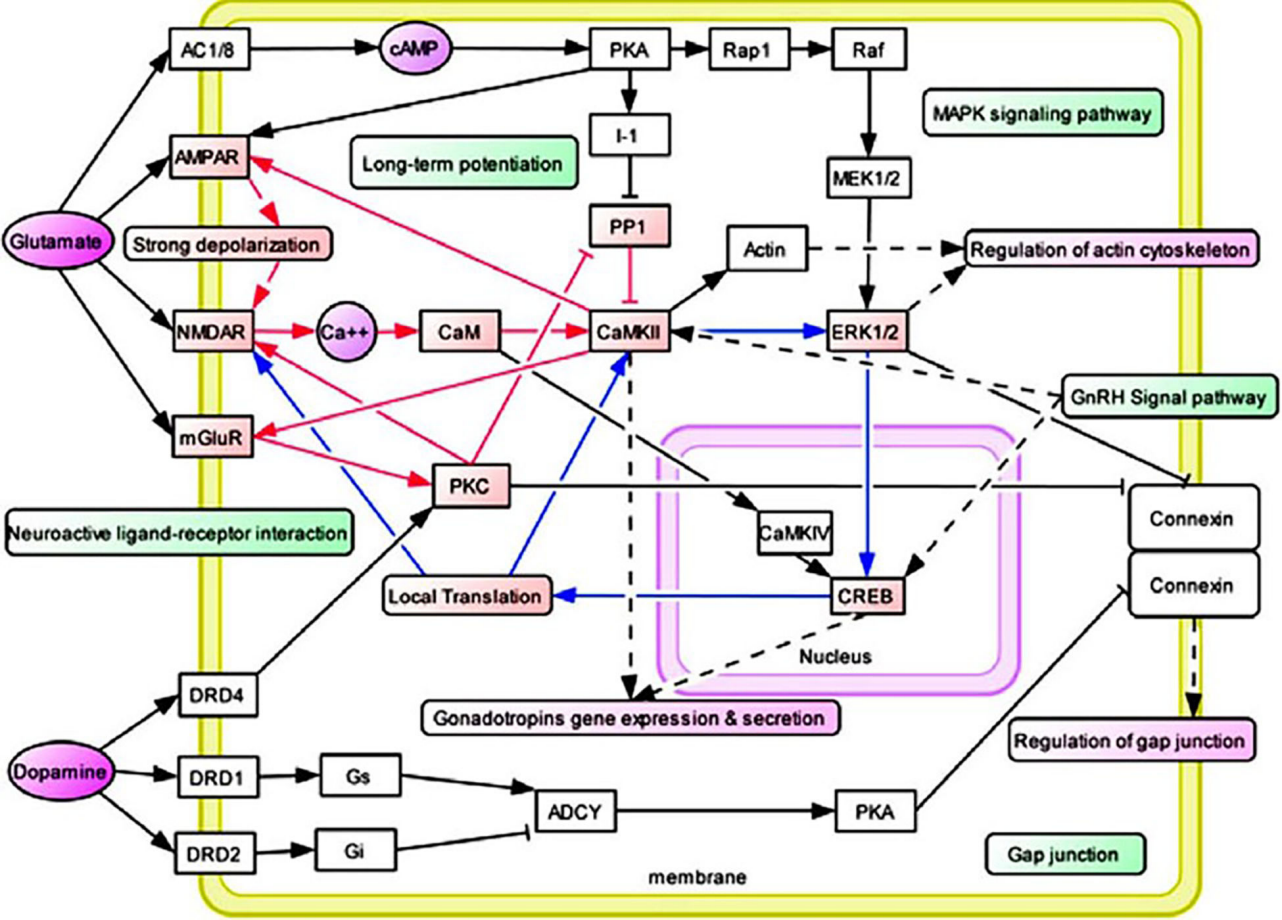
Hypothetical common molecular network for drug addiction.

Neurofeedback training’s ability to self-regulate GABA activity, known to reduce dopamine release at the NAc, suggests its role in promoting a physiologically healthy brain. When viewed in the context of dopamine modulation, GABA control may contribute to a “happy brain” state (Figure 6 and figure 7) [260, 261].

A recent study succinctly demonstrated that electrically stimulated neurons triggered greater DA release in the NAc of 3-KO mice with better reward learning and even decision-making [245] if the gene that codes for the three subunits of the GABA(A) receptor is knocked out (KO). Notably, GABA-evoked GABA-gated inhibitory postsynaptic currents in dopamine neurons in midbrain slices from 3-KO mice were reduced (IPSCs). Additionally, electrical stimulation of excitatory afferents to dopamine neurons caused an increase in DA release in the NAc of 3-KO mice, as observed by fast-scan cyclic voltammetry. When fed morphine, 3 KO mice were more active than control mice. This effect was attributed to a putative upregulation of GABAergic tone onto dopamine neurons as a compensatory mechanism. 3-KO mice learned more quickly in two food-reinforced learning paradigms but generally lost their new behaviors. Aversive learning was unchanged in 3-KO mice, indicating that the enhanced learning was specific to appetitive activities. Moreover, Blum et al. [259] discovered that in a probabilistic selection test where mice had to choose between a smaller known incentive and a greater uncertain reward, 3-KO mice showed enhanced risk preference. Collectively, these results, according to the authors, point to a specific function for GABA (A) signaling in dopamine neurons in the learning and decision-making associated with appetite [261, 262].

### Transcranial magnetic stimulation

rTMS is a noninvasive brain stimulation technique currently approved for major depressive disorder. rTMS is currently being pursued as a treatment for SUD. Preliminary data looking at treatment with rTMS to the dorsolateral PFC (DLPFC) has shown a reduction in cravings in alcohol, cocaine, and nicotine use disorders. Single-session rTMS studies have demonstrated that applying excitatory rTMS to the DLPFC can decrease cue-induced craving in nicotine, cocaine, and AUD populations [263–267]. The mechanism of rTMS success in treating addiction is thought to involve increased dopamine function in the shell region of the NAc. The largest clinical trial (n = 130 smokers) demonstrated that 13 sessions of DLPFC rTMS resulted in abstinence rates of 33% in six months. To date, there is limited work examining the effect of rTMS on craving in OUD [264, 266, 267], but personalized rTMS (PrTMS) may have significant value in the future.

### A novel approach to pain management

The resolve to shift the drug-embracing culture in American chronic pain management away from the reliance on opioids is imperative, especially given the weak evidence supporting the long-term use of opioids for pain relief. Non-addictive, non-pharmacological methods exist to effectively manage chronic pain without resorting to opioids, despite the opioid overdose epidemic. Unfortunately, some pain management doctors still favor traditional analgesics, which not only encourage unwelcome drug tolerance but also biologically induce addictive brain pathways [267]. Chronic opioid use can lead to addiction, drug tolerance, neuroadaptation, hyperalgesia, and potentially addictive behaviors, all contributing to RDS characterized by hypodopaminergia [268].

A meticulous analysis revealed that GARS scores equal to or greater than 4 and 7 alleles significantly predicted drug and alcohol severity, respectively, among pain clinic patients. By utilizing the GARS test and the Addiction Severity Index (ASI-Media Version V), Wise et al. conducted a study involving 121 chronic opioid users. Their findings underscored a significant hereditary risk of opioid and alcohol addiction among chronic opioid users receiving legal prescriptions and visiting pain clinics [269]. Employing the GARS test at treatment onset to identify genetic risk early may help mitigate iatrogenic opioid dependence.

It is prudent to cautiously explore the potential of identifying reward circuitry gene polymorphisms associated with dopaminergic pathways and opioid receptors to enhance treatment outcomes [270]. Notably, research by Gardner’s team at NIDA demonstrated that decreased D3 receptor availability in the brain is a risk factor for opioid abuse and addiction, as evidenced by dopamine D3 receptor-knockout (D3-KO) mice [162].

While the study primarily focused on DNA polymorphisms, it is crucial to recognize that opioids exert epigenetic effects on mRNA transcription and the genetic expression of these risk alleles. Hence, future research should explore potential protective mechanisms in individuals without DNA polymorphic risks [271]. Employing the GARS test upon entry into a pain clinic can assess the risk of opioid-induced dependence, aiding in personalized treatment approaches. Additionally, investigating how race and gender influence opioid dependence risk could provide further insights into personalized pain management strategies [123, 272–278].

Given that adaptation in dopaminergic circuitry and nociception regulation heavily depend on reward genes, employing the GARS test alongside modalities like H-Wave or rTMS at entry into pain clinics could mitigate discomfort and prevent addiction. Identifying high-risk individuals for alcohol and drug addiction through the GARS test allows for the initiation of interventions like H-Wave for pain relief instead of opioids, thereby reducing the risk of fatalities and long-term neurological abnormalities associated with potent opioid analgesics. Despite the need for randomized control studies, incorporating H-Wave to alleviate pain and mitigate addictive behaviors proactively is advised.

### The utility of cannabinoids/psychedelics as risk-reduction tools

In a JAMA article reviewed by Dr. Peter Grinspoon, MD, from Harvard Medical School, a longitudinal analysis of opioid prescriptions filled under Medicare Part D revealed that the implementation of medical marijuana laws in a state led to a reduction of 2.21 million daily doses of opioids filled per year. Additionally, with the opening of medical marijuana dispensaries, opioid prescriptions decreased by 3.74 million daily doses per year. These reductions were particularly pronounced for opioids like hydrocodone (Vicodin) and morphine but also extended to benzodiazepines, stimulants, and various mental health medications known for over-prescription and harmful adverse effects. Dr. Yasmin Hurd elaborated on this topic in a comprehensive 2017 article, proposing an evidence-based approach to addressing the opioid epidemic by leveraging the endocannabinoid system, with a focus on non-psychoactive cannabinoid molecules like cannabidiol (CBD) [278].

Recognition of the significance of the endocannabinoid system has grown within the scientific community, offering insights into chronic illnesses such as chronic intractable pain and other difficult-to-treat conditions that often lead to excessive opioid prescriptions. While cannabinoids, particularly CBD, generally exhibit a favorable safety profile [279, 280], it is essential to acknowledge potential side effects such as sleepiness, diarrhea, and changes in appetite/weight. However, unlike opioids, there have been no reported deaths attributable to CBD use.

Despite the established safety profile, many clinicians lack education on cannabinoids and the endocannabinoid system [281,282], leaving patients to self-medicate with cannabinoids in experimental ways [283]. This includes instances of overuse of THC-dominant cannabinoid products, which carry an increased risk of adverse effects such as drowsiness, lack of motivation, hyperphagia, and, in some cases, anxiety and paranoia. Notably, most adverse effects associated with cannabinoid products stem from incorrect dosing, mislabeled products, and occasionally contaminated products [284–288].

Similar to other neuropsychiatric disorders, addiction is influenced by pathological neuroplasticity, resulting in structural and functional changes in brain networks that contribute to maladaptive behaviors. Psychedelic drugs like ketamine, ibogaine, psilocybin, MDMA, LSD, and ayahuasca have shown promise in therapeutic settings for treating addiction, PTSD, depression, and anxiety. Through serotonin 2A (5-HT2A) receptor agonism, psychedelics induce mind-altering effects that may “reset” brain regions involved in various neuropathic conditions. Studies have demonstrated the effectiveness of psychedelic-assisted therapy in nicotine cessation, depression, and anxiety, providing individuals with a sense of connection and spiritual experience that addresses the underlying void they seek to fill [289–297]. Further research is warranted to explore the potential of ibogaine in treating opioid addiction.

### Psychological

Individuals grappling with OUD and co-occurring trauma face profound challenges, including overwhelming emotional distress, a sense of personal disempowerment, and feelings of disconnection from others and their surroundings. Within trauma recovery circles, it’s recognized that many individuals experiencing addiction inhabit what is metaphorically referred to as the “Realm of Hungry Ghosts” [189].

It’s essential to acknowledge that individuals in this state of perpetual struggle often contend with a chronic lack of psycho-social-spiritual integration, manifesting as a profound sense of disconnection from others. This disconnection can lead to feelings of isolation and may be linked to schizoid-avoidant behaviors associated with DRD2 polymorphisms [290]. Given the complexity of these issues, there is no one-size-fits-all approach to treatment. However, there is a notable convergence of common neurochemical, genetic, and epigenetic factors underlying all RDS behaviors, encompassing both substance and non-substance behavioral addictions [291, 292].

Effective trauma-informed treatment necessitates a deep understanding of patients’ multifaceted presentations and the enduring impact of personal traumatic experiences on their lives. This awareness should form the cornerstone of treatment approaches. All individuals with OUD grapple with a common adversary: the recurring self-blame that reinforces the belief that they alone are responsible for their addiction. This cycle of self-loathing thrives within the repetitive thought patterns characteristic of ongoing addiction and, without intervention, can intensify over time, undermining treatment efforts and increasing susceptibility to relapse [292–294].

### AIT

AIT represents a multi-modality psychotherapeutic paradigm designed to augment self-awareness, alleviate past traumas and psychological barriers, and foster clarity and positive attitudes. Constructed by amalgamation of insights and techniques drawn from diverse therapeutic models such as Cognitive Behavioral Therapy (CBT), Existential Therapy, Person-Centered Therapy, Emotion-Focused Therapy (EFT), Mind-Body Therapy (MBT), Eye Movement Desensitization and Reprocessing (EMDR), hypnosis, and mindfulness, AIT offers a comprehensive approach. By integrating various elements from these methodologies, AIT establishes an inclusive and adaptable framework to address the entire human experience. This ensures the optimization of therapeutic efficacy, enabling the generation of lasting and transformative outcomes for individuals undergoing treatment [295].

AIT aims to reconcile fragmented aspects of the “Self” from psychological trauma and facilitate self-awareness spanning from past experiences to the present [296]. This model embraces an individual’s life journey through an effective, and open-ended approach. It empowers patients to explore and comprehend the interplay between their perception of the environment, interactions with others, and behavioral patterns using specified awareness skills. By delving into conscious and subconscious processes, AIT enables the reevaluation of irrational thoughts and emotions while assisting clients in developing valuable life skills [296].

The AIT model provides systematic guidance for identifying fragmented aspects of the self and integrating them to function harmoniously and productively. Through this multistep process, patients discern both constructive and non-constructive thoughts and mental schemas, thereby identifying ingrained fundamental beliefs, emotions, and behaviors. Subsequently, patients learn new strategies to replace negative beliefs with constructive and beneficial principles. As AIT explores the interconnectedness of the self with various life domains, including relationships, family dynamics, career, finances, and spirituality, transformations extend across these realms [297].

Approaches that solely focus on the biological detoxification of individuals with OUD while neglecting psychological and emotional healing are inherently flawed [298]. Conversely, treatments that comprehensively assess neurobiological factors and address motivation for change, skill development, environmental influences, family dynamics, and social context are more likely to succeed. Short-term interventions often yield limited results, whereas long-term treatment, incorporating detoxification, environmental modifications, and ongoing work on beliefs, emotions, and maladaptive behaviors, offers greater success. An effective treatment plan should include relapse prevention strategies and equip patients with awareness skills to recognize warning signs before relapse occurs. It is imperative that treatment interventions encompass all facets of an individual’s life, encompassing both interpersonal and intrapersonal dimensions. AIT acknowledges the individual’s relationship with their substance of choice and their relationships, work, finances, and self-identity [298].

For substance abusers, the prospect of life without their preferred narcotic seems unimaginable as addiction often serves as a substitute for love, connection, energy, and joy [299]. Motivating change in such individuals can occur through either embracing a more fulfilling alternative or facing the consequences of continued substance use. However, relying solely on avoidance of pain often leads to existential resentment and depression, perpetuating a cycle of relapse. Love and acceptance can serve as powerful catalysts for initiating sobriety, yet sustained recovery requires individuals to engage in internal work to address underlying emotional turmoil. The AIT empowers individuals to evaluate their thoughts, feelings, and behaviors, fostering self-awareness and facilitating meaningful change, even amidst genetic predispositions and epigenetic influences [300].

To live with a person with an addiction of any kind is frustrating, painful, and emotionally draining. Therefore, no matter how much the family of an addict loves and cherishes them, at one point, their boundaries and judgments will show up, and the person with an addiction will take that as rejection. This perception of rejection will fuel their shame and turn them right into using it again. Therefore, AIT as a psychotherapeutic approach that offers unconditional acceptance and love while allowing the person with an addiction to become aware and accountable for their choices and results in a non-judgmental way motivates them to stay on the path of recovery even if they relapse. If the individual with addiction is married, has children, or lives with parents, additional family psychotherapy is suggested to facilitate conversations, conflict resolutions, and boundary settings among the family members. With the AIT process, family members can also become aware of their behaviors and their impact on the person with an addiction. Each family member can become responsible and accountable for how they have treated the person with an addiction, enabled, used, abused, nurtured, set boundaries, or lack thereof [296–301].

AIT has demonstrated efficacy in addressing childhood traumas and reducing rates of depression and anxiety among COAs. Most individuals with substance dependence, as well as those without, often originate from dysfunctional family backgrounds, with their addictions exacerbating the existing dysfunction. Genetic testing of such families would likely reveal a high genetic predisposition to addiction based on reward gene polymorphisms. Children of Alcoholics (COAs), for instance, represent a special population group that could benefit from early genetic screening due to their heightened risk. Claudia Black introduced the concept of COAs in her work, highlighting the profound impact of dysfunction and stress within their homes [301]. COAs commonly endure depression, anxiety, and social difficulties, lacking secure role models to navigate life’s challenges. Studies have shown a significant association between COAs and the DRD2 A1 allele, underscoring the genetic vulnerability within this population [297, 302]. By addressing the absence of healthy parental role models, AIT equips adults with essential life skills for creating functional and fulfilling lives. Moreover, AIT supports the development of healthier family dynamics and interpersonal relationships. Children have also benefited from AIT by learning coping mechanisms and emotional regulation skills to navigate the complexities of a chaotic family environment [303].

Adolescent depression and anhedonia are prevalent yet often overlooked and untreated. Approximately one in five children may experience emotional, behavioral, or mental health issues, with a significant proportion suffering from mild to severe depression. However, only a fraction of affected adolescents receives adequate treatment, leaving many vulnerable to substance abuse, risky behaviors, poor academic performance, and even suicide [304]. Furthermore, adolescents with OUD may lack essential life skills such as emotional regulation, communication, and financial management. AIT offers strategies for building these skills, empowering adolescents to lead fulfilling lives while fostering healthy relationships [305]. Collaborative efforts between AIT practitioners and families facilitate a cohesive emotional and behavioral regulation approach, providing crucial support and guidance to adolescents [301]. Given the frequent comorbidity of addiction with mood, anxiety, PTSD, and personality disorders, long-term weekly psychotherapy is imperative. Research on AIT has shown promising outcomes, including significant reductions in depression and anxiety levels, along with improvements in self-esteem and self-efficacy [297].

### AIT 6 phase intervention

Phase 1 is initiated by fostering an awareness of the participant’s cognitive processes, emotional responses, and behavioral patterns concerning their external environment and elucidating how these constructs influence their daily experiences. This phase entails posing questions to probe participants’ perceptions and attitudes toward others. This initial phase of therapy often unveils prevalent generalized belief systems held by the individual, shedding light on foundational cognitive structures that shape their worldview and interpersonal interactions [295].

Phase 2 serves three essential functions: 1) Facilitating the individual’s awareness of their subjective projections regarding others’ opinions and perceptions of them. 2) Strengthening the participant’s awareness of the internal processes in attributing meaning to these perceptions. 3) Evaluating the impact of these internal projections on the individual’s life and cultivating skills to validate these perceptions against external reality. During this phase, clients are prompted with inquiries designed to uncover their assumptions regarding how others perceive them. This phase proves particularly influential for individuals grappling with heightened levels of anxiety and social phobia, as it offers a structured approach to examine and recalibrate their perceptions of interpersonal interactions [296].

Phase 3 represents a pivotal stage, fostering the individual’s reflective understanding of their identity. This phase is paramount in the therapeutic process, aiming to elucidate the participant’s core beliefs. Specifically, the following questions are posed to the participant: How do you perceive yourself when you are among others, observing their reactions to you? What emotions do you experience regarding your self-perception? How do you treat yourself in your thoughts, feelings, and actions? Do you engage in self-judgment? Do you demonstrate self-compassion? How do you assess your overall well-being? These inquiries are carefully crafted to delve into the participant’s foundational beliefs about themselves and their self-image [296].

Phase 4 is characterized by a structured approach aimed at exploring the interconnectedness between thoughts, cognitive patterns, emotional responses, and bodily sensations, particularly the origination of negative core beliefs stemming from traumatic experiences. During this phase, the focus is on examining irrational thoughts and decision-making strategies. At the same time, the individual engages in self-assessment regarding how negative thoughts and emotions impede rational and logical thought processes and learns the skills to manage emotions effectively. This phase encourages participants to delve into the original memory associated with the felt sensation and bodily responses, facilitating release, healing, and integration of fragmented or dissociated aspects into the holistic self. This process fosters a re-evaluation of self-belief systems, associating new emotions and behaviors with the self, thereby fostering enhanced connectivity with the external world [296].

Phase 5 is a proactive step focused on guiding participants through visualization exercises and commitment toward cultivating a new and improved self-concept. Emphasis is placed on nurturing a positive attitude and fostering a sense of agency in shaping one’s perceptions and actions towards oneself and others. Questions in this phase are tailored to explore individual values and beliefs, prompting participants to contemplate their intended identity and desired thought patterns, emotions, and behaviors. The success of this phase is contingent upon the participant’s commitment to implementing AIT strategies, thereby anticipating personal growth and achievements [296].

Phase 6, the final AIT intervention step, entails structuring a functional value system encompassing intentions, emotions, and behaviors conducive to everyday life. Participants are encouraged to design their desired positive self, which can be externalized through visual aids such as collages, providing consistent reassurance and guidance towards realizing their aspirations [296].

### Proposed AIT intervention for treatment of SUD

Session 1: Overall History Interview (OHI) from the patient and family members.

Session 2: Phase 1, 2, and 3 in the area of addiction.

Session 3: Phase 4, in the area of addiction.

Session 4: Phase 1, 2, 3, and 4 in the area of job/career.

Session 5: Phase 1, 2, 3, and 4 in the area of finances.

Session 6: Phase 1, 2, 3, and 4 in the area of intimate relationship/marriage.

Session 7: Phase 1, 2, 3 and 4 in the area of relationship with children.

Session 8: Phase 1, 2, 3 and 4 in the area of relationship with siblings.

Session 9: Phase 1, 2, 3 and 4 in the area of relationship with parents.

Session 10: Phase 1, 2, 3 and 4 in the area of relationship with body.

Session 11: Phase 1, 2, 3 and 4 in the area of spirituality and death.

Session 12: Phases 5 and 6 goal setting and structured action plans in all areas of life.

Follow-up sessions will prioritize assisting the client in achieving their objectives, overcoming barriers to sobriety, and striving for balance across all facets of life. Given that addiction affects various aspects of an individual’s life, the treatment approach should similarly encompass all these dimensions. Relapse prevention will be facilitated through balancing and creating fulfillment in all areas of life.

### CBT

Recent systematic reviews have raised doubts about the efficacy of psychosocial interventions in augmenting buprenorphine therapy significantly. However, CBT is often touted as an effective treatment for OUD, taking into account various factors such as the severity of addiction, living environment, skill level, past traumas, emotional resilience, and family support. Extensive evidence supports the positive clinical outcomes associated with CBT. Gregory and Ellis conducted a meta-analysis to test the null hypothesis within the random effects model (REM), which posited that the summary effect of CBT + buprenorphine randomized controlled trials (RCTs) for OUD and opioid biological sample outcomes equals zero, thus indicating the absence of an effect. However, the initial meta-analytic model yielded insignificant results. Nevertheless, the disparity between these two subgroups was statistically significant. While not definitive, the evidence tentatively suggests that the addition of non-individual CBT to buprenorphine therapy may confer some benefits for individuals with OUD [306].

### Mindfulness

Research indicates that incorporating mindfulness into treatment plans for complex trauma and addiction can be highly effective [307–310]. In cases where trauma and addiction intersect, treatment strategies should include mindfulness interventions tailored to address trauma sensitively. Mindfulness, when approached with trauma sensitivity, helps individuals disengage from the repetitive thought patterns associated with addiction. It empowers them to regain control over addictive thoughts and actively participate in their recovery journey, which is often lifelong.

It’s important to recognize that mindfulness practices emphasize experiential acceptance and present-moment awareness, relying on attention regulation, self-awareness, and emotion regulation skills. While further research on mindfulness is warranted, preliminary findings are beginning to shed light on its neural correlates. For instance, Sezer et al. conducted integrative studies on functional connectivity, trait mindfulness, and mindfulness meditation interventions [311]. They found that mindfulness practices are associated with functional connectivity changes in key brain networks, including the DMN, frontoparietal network (FPN), and salience network (SN). These changes encompass improved communication between brain regions involved in attention management, self-awareness, emotion regulation, and pain alleviation, indicating emerging neural signatures of mindfulness.

Mindfulness meditation training has been shown to enhance the resting-state functional connectivity between the frontoparietal executive control network, particularly the DLPFC, and the DMN, specifically the posterior cingulate cortex (PCC). Kral et al. [295] observed a heightened T2-T1 PCC-DLPFC resting connection in participants who underwent mindfulness-based stress reduction (MBSR) compared to control groups following mindfulness training. Notably, mindfulness individuals exhibited a significantly stronger connection between practice days (T1 to T3) and enhanced PCC-DLPFC resting connectivity compared to those in the active control group. Furthermore, improvements in microstructural connectivity of the white matter tract linking these regions and self-reported attention enhancements were associated with increased PCC-DLPFC resting connectivity among mindfulness practitioners. These findings suggest that mindfulness practice positively impacts the PCC-DLPFC resting connection. Mindfulness presents an opportunity for individuals grappling with trauma and addiction to reclaim control over their thoughts and emotions, fostering a sense of calm. However, given the complexity of trauma, mindfulness alone may not suffice. Best practices advocate for a trauma-sensitive approach to mindfulness, recognizing its role as a valuable component of treatment and recovery. Therefore, the operative term regarding trauma-sensitive mindfulness is ‘caution’ [298].

Further research into mindfulness, conducted by Davidson’s team, has revealed the importance of meditation in activating the brain’s reward system. Expert meditators exhibited greater activation in attention and impulse control tasks, such as the Stroop Word-Colour Task (SWCT), compared to novices. This indicates that meditation and enhanced spiritual conviction may lead to dopamine release in areas like the VTA and cingulate gyrus, potentially improving clinical outcomes and reducing relapse rates [295, 312]. The U.S. National Center for Trauma-Informed Care has outlined a guideline, known as the ‘Four R’s,’ to inform trauma-sensitive mindfulness practice as part of a comprehensive recovery plan [295]:

- Mindfulness practice acknowledges the widespread impact of trauma and acknowledges potential pathways to recovery.
- Mindfulness practice identifies signs and symptoms of trauma in individuals.
- Mindfulness practice integrates knowledge of trauma into the practice and response.
- Mindfulness practice safeguards against re-traumatization.

Just as individualized treatment plans cater to each patient’s unique needs, trauma-informed mindfulness practice meets patients where they are and respects their boundaries. Trauma-informed care endeavors to establish a safe environment where individuals in recovery can explore their complex trauma and addiction challenges with a sense of security.

### Spirituality

A blended treatment approach to trauma and addiction must encompass diverse strategies to create sensitive pathways for individuals grappling with addiction to address underlying traumas and develop resilience. One of the primaries aims of such treatment is to restore psycho-social-spiritual integration and balance [313–318].

Substance addictions and non-substance behaviors such as gambling and internet gaming disorders are pervasive worldwide, leading to adverse health outcomes, criminality, and societal productivity loss. Genetic vulnerability, compounded by environmental factors, contributes to biochemical imbalances and brain dysfunction underlying addiction. Given the compulsive nature of addiction, it poses significant challenges for study and treatment. We hypothesize that understanding the role of spirituality in healing dependency disorders may offer valuable insights. Moreover, genetic engineering, or “Geno spirituality,” has the potential to enhance human spiritual and religious experiences, albeit receiving limited attention thus far [319].

The medical applications of genetic engineering have been widely debated, with discussions extending to its potential as a tool for spiritual enhancement. Geno spirituality could involve modifying genes responsible for spiritual and religious inclinations, allowing individuals to select their religiosity or spiritual sensitivity levels safely and conveniently. This could potentially enable individuals to experience direct religious revelations or foster animistic thinking, enhancing their spiritual connection with the world. Shamanism, characterized by altered states of consciousness and contact with a spirit realm, presents another avenue for spiritual enhancement [320].

An ideal state of shamanistic consciousness would allow individuals to induce trances at will, ensuring safety and practicality while maintaining the ability to return to full alertness as needed. However, the pursuit of spirituality may entail costs, such as reduced desire for material gain, potentially influencing work habits and leisure activities. Conversely, heightened spirituality could lead to increased moral, altruistic, and principled behaviors, fostering stronger community bonds and well-being [321].

**Figure 8:**
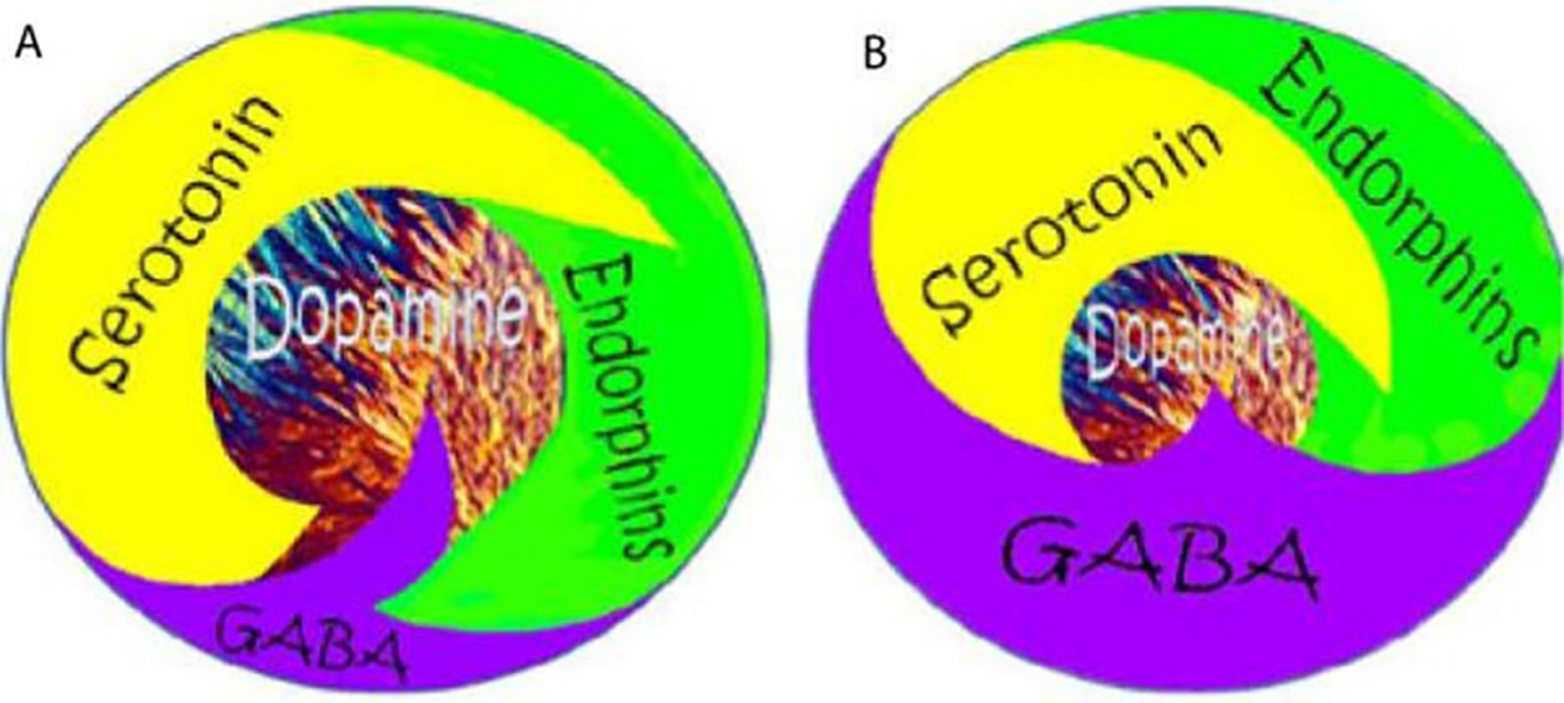
Brain reward cascade (normal and hypodopaminergic state) [with permission Blum et al.] **8(A) Happy brain:** Represents the mesolimbic area of the brain’s typical physiologic condition for the interaction of neurotransmitters. In summary, serotonin activates the neuronal projections of methionine enkephalin in the hypothalamus, which suppresses the release of GABA in the substania nigra and promotes the release of the normal amount of dopamine in the NAc, the brain’s reward center [260]. **8(B) Unhappy brain:** Represents the mesolimbic area of the brain’s hypodopaminergic activity. Environmental factors, such as stress and neurotoxicity from abnormal consumption of psychoactive drugs (such as alcohol, heroin, cocaine, etc.,), hereditary factors, and gene polymorphisms, all contribute to the hypodopaminergic state [260].

**Figure 9:**
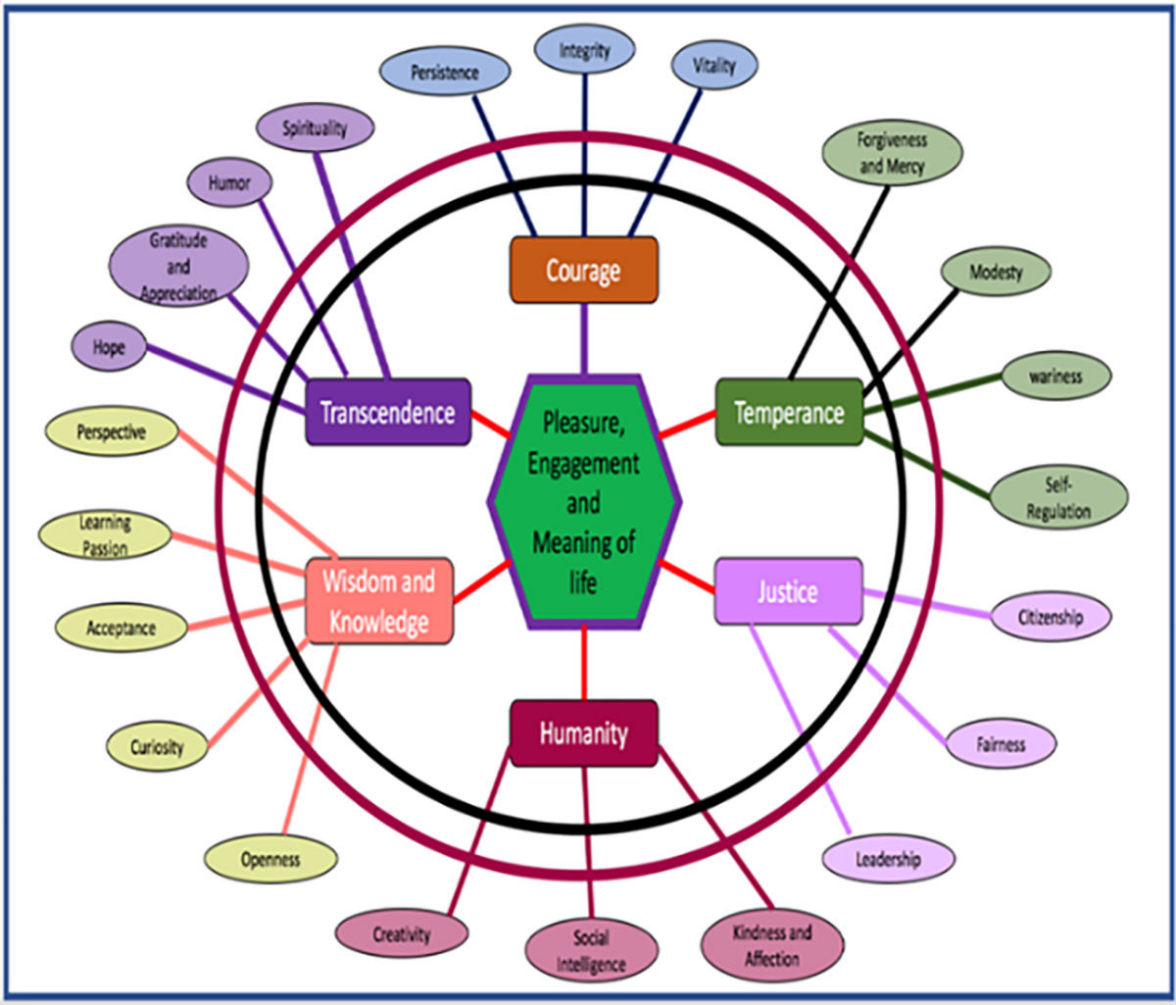
Characteristics of life’s meaning modified from 538.

To support these concepts, we propose the Neurospiritual Connectome, which we suggest underlies the Purpose and Meaning of Life as Reward (PMLR), potentially mitigating the effects of untreated addictions figure 8 concerning happy brain vs unhappy brain. Additionally, seminal work by Seligman and others underscores the importance of nurturing well-being alongside treating mental illness. Positive psychology emphasizes building positive emotions, gratification, and meaning in life to enhance overall happiness and quality of life (Figure 9).

Most recently, Li and colleagues developed an addiction gene network manually constructed based on the common pathways identified in their 2008 study and protein interaction data. In the network, addiction-related genes were represented as white boxes, while neurotransmitters and secondary messengers were highlighted in purple. Common pathways were depicted in green boxes, while related functional modules such as “regulation of the cytoskeleton,” “regulation of the cell cycle,” “regulation of gap junctions,” and “gene expression and secretion of gonadotropins” were highlighted in carmine boxes. Several positive feedback loops were identified within this network, with fast positive feedback loops highlighted in red lines and slow ones in blue (with permission).

In summary, a holistic approach to addressing trauma and addiction involves integrating diverse strategies, including exploring the role of spirituality and potential advancements in genetic engineering to enhance spiritual experiences. Moreover, fostering well-being alongside treating mental illness is crucial for promoting overall happiness and life satisfaction.

### Efficacy of 12-step programs

A crucial component of the recovery journey involves the support of a nurturing, spiritual community such as alcoholic/narcotic anonymous or Al-anon for families, complemented by outpatient group programs and individual and family psychotherapy. These communities offer unconditional support and a sense of belonging, providing continuous availability compared to therapists and psychiatrists, who have limited availability and higher costs. Having a spiritual community to lean on during moments of craving or emotional turmoil is essential for sustaining individuals through the recovery process [322].

Many individuals grappling with addiction achieve abstinence successfully upon enrolling in a treatment program or participating in the 12-Step Program and Fellowship. When conventional methods like regulated drinking prove ineffective, tailored solutions tailored to specific demographic groups may yield success. Alcoholics Anonymous (AA), a worldwide mutual support organization, was founded in Akron, Ohio, in 1935 by Bill Wilson and Dr. Bob Smith (Bill W. and Dr. Bob). The primary mission of AA is to inspire alcoholics to maintain sobriety and extend assistance to others striving for sobriety. Wilson, Smith, and early members devised the Twelve-Step spiritual and character development program, which serves as the cornerstone of AA’s approach. In 1946, the Twelve Traditions were introduced to foster the growth and sustainability of AA. These Traditions advocate for anonymity in public settings, inclusivity of all seeking sobriety, and the altruistic support of fellow alcoholics. Furthermore, the Traditions recommend that AA members refrain from embracing specific ideologies, engaging in political activism, or establishing governing structures when representing the fellowship. Over time, the Twelve Steps and Twelve Traditions have been adapted and adopted by subsequent organizations to suit their unique missions, as evidenced by groups like Narcotics Anonymous (NA) [323].

AA typically refrains from delving into the medical aspects of alcoholism; however, it is often credited with popularizing the illness hypothesis of alcoholism [14]. For chronic alcoholics resistant to brief therapy, the American Psychiatric Association has recommended the AA program alongside similar community options and ongoing treatment. According to AA statistics, 64% of new members discontinue participation within the first year [324, 325].

Since its inception in 1935, AA has broadened its membership to encompass individuals from diverse cultures with distinct viewpoints and values, particularly in regions where grassroots movements have faltered. Membership in AA reportedly exceeds 2 million individuals. While the 12-step program and the AA/NA fellowship may exhibit some variances, both can play integral roles in an individual’s recovery journey. The organization derives its name from the seminal work “AA: The Story of How More Than One Hundred Men Have Recovered from Alcoholism,” also known as the Big Book. Contrary to the notion that individuals with a genetic predisposition to ethanol allergy are destined for a life of addiction, recent insights into neuroepigenetics underscore the impact of environmental factors on polymorphic genes, particularly those linked to the brain’s reward circuitry. Although no universal remedy exists, and while many individuals may find solace in 12-step programs, are we edging closer to unraveling the complexities of addiction [326]?

Clarifying the molecular neurobiological mechanisms underlying each step of the 12-step program enhances our comprehension of individuals’ diverse approaches toward recovery. Investigating the effects of these neurobiological underpinnings on RDS despite addiction risk gene polymorphisms is a worthwhile pursuit. Of particular interest is exploring the potential influence of epigenetic modifications in individuals who regularly attend AA/NA meetings. While further extensive research is necessary, it is worth considering the hypothesis that participation in “12-step programs and fellowships” may facilitate neuroplasticity and the continual expansion of dopamine D2 receptors, even in individuals with hypodopaminergic polymorphisms like the DRD2 A1 allele. Notably, Blum’s team has demonstrated the induction of “dopamine homeostasis” in the brain’s reward circuitry through the manipulation of seven neurotransmitter systems, presenting a potential avenue for intervention [327]. Integrating dopamine agonist modalities (DAM) as potential histone-deacetylase activators with the 12-step program or medical-aided therapy (MAT) warrants further exploration, although unresolved questions remain. Nonetheless, the convergence of science and recovery heralds promising prospects for restoring happiness in individuals undergoing recovery.

The neurobiological foundations of the 12 steps and similar programs such as Al-Anon underscore the importance of grasping these concepts as integral components of achieving and sustaining sobriety. Adopting molecular neurobiology principles may contribute to an improved quality of life during recovery. Evidence suggests that participation in the 12-step program and fellowships facilitates enhanced communication between brain regions involved in decision-making (PFC-Cingulate) and craving behavior NAc, shedding light on the neurogenetic basis of addiction and the human pursuit of happiness, elucidated through decades of dedicated scientific inquiry into the mesolimbic system [328].

### Controversy

Harvard Professor George Vaillant found no proof of the AA program’s effectiveness compared to a control group not utilizing the AA/NA program. Vaillant concluded: “AA may be a good and comfortable fit for a few people who have a problem with alcohol, but most people with alcohol problems appear to do better with a different approach. We would love to see a study of why so many people dropped out of AA. We hypothesize that this may be because’ AA’s theological notions of the powerlessness of humanity and the need for a rescuing God are unpalatable not only to many atheists and agnostics but to almost all theists who are not Calvinists as well”. Furthermore, Vaillant suggests based on his research that: “It may also be the case that the AA philosophy of “powerlessness” over alcohol and slogans such as “one drink, one drunk, “one is too many and a thousand is never enough” and “alcohol is cunning, baffling, and powerful” set people up to binge drink rather than to practice damage control when they slip up and fail to abstain as intended [328].

Vaillant summarizes reasons AA [NA] doesn’t function for everyone [237]. AA is a good fit and encourages abstinence only for a small percentage of individuals with alcoholism and not everyone. AA cultivates “true believers” rather than eradicating problem drinking. The critical criterion in determining whether AA is a suitable fit for a person is their personality type. Black-and-white thinkers who accept proof of authority will fit in well with AA; however, those who need experimental evidence and scientific validation perceive things as having gray zones that won’t be helped. In the future, conducting genetic testing to determine if a person is more likely to adopt AA teachings can be helpful [315].

Interestingly, the “why AA does not work” search term in a PUBMED search (9–5-14) produced no hits. However, Kelly [322] made the following points: “Regarding subpopulations, current evidence suggests non- or less-religious individuals benefit as much from self-help groups as more religious individuals and women become as involved and benefit as much as men. However, participation in, and effects from, traditional self-help groups for dually diagnosed patients may be moderated by type of psychiatric comorbidity. Some youth appear to benefit but remain largely unstudied. Dropout and nonattendance rates are high, despite clinical recommendations to attend.”

Since Vaillant assessed the 12 steps in relapse reduction, several findings have emerged, contradicting earlier notions and emphasizing the roles of spirituality, transcendence, personality, and meditation. Blum’s team discovered that increasing belief in spirituality correlates with reduced relapse rates [329]. Research exploring spirituality from a neurotransmitter perspective has yielded mixed results. Finnish researchers found no link between spiritual experiences and 5-HT-1A receptors in major depressive patients or healthy controls [329]. Conversely, Borg et al. found a significant correlation between spiritual acceptance and the presence of 5-HT-1A receptors across various levels [329]. This suggests a potential connection between spiritual fervor and neurobiology. Individuals with specific gene variants, such as homozygosity for the long AP-2beta gene and the short 5-HTTLPR, scored lower on self-transcendence and spiritual acceptance scales [330].

Genetic factors undoubtedly influence behavioral addictions and SUD, with both RDS subtypes appearing to be heritable. RDS is a complex, polygenic disorder with numerous epigenetic effects on DNA chromatin structure and function. Belcher et al. identified gene polymorphisms associated with three high-order personality traits, which likely influence brain function and, consequently, personality and belief systems [331]. These genetic predispositions may render individuals more susceptible or resistant to developing OUD.

While many treatment centers endorse the 12-step program and its helper principle, its efficacy as the sole treatment option has been questioned. Despite reservations about medication-assisted therapy (MAT) within AA/NA circles, Chappel and Dupont advocated for its integration, particularly for individuals with co-morbid psychiatric conditions [332]. They stressed the importance of involving friends and family in network therapy to prevent relapse, echoing Galanter’s group’s findings [333]. Teitelbaum also emphasizes the role of family in addiction prevention [344].

Galanter et al. found that patients with a greater spiritual inclination, as opposed to religiosity, exhibited reduced substance-seeking behavior, highlighting the potential efficacy of spiritual interventions [173]. Clinicians are encouraged to stay abreast of molecular neurogenetic literature, especially as it pertains to the reward system and addictive behaviors. Of particular concern are individuals who have relapsed after rehabilitation and engaged in criminal behavior to obtain illicit opioids [335].

### Overall treatment suggestions

Based on the above extensive discussions, the following treatment options are suggested:

1. Internists, family medicine, and psychiatrists to evaluate and screen for drug use/abuse or dependence.
2. Brain screening is to be offered to assess medication management and possibly behavioral modification appropriately.
3. With continued research, utilization of GARS testing, or any other genetic screen is needed to identify DNA risk antecedents in patients and families.
4. Inpatient detox facility to detox the patient if necessary, or outpatient detox via methadone or suboxone for a short period, then switching to more gentle pro-dopamine regulation (e.g., KB220 variants).
5. Residing 3 – 12 months at a sober living home offer a supportive environment conducive to recovery, allowing individuals to develop emotional regulation, communication, and negotiation skills necessary for initiating positive changes in their family dynamics.
6. Medical treatment under the care of a psychiatrist with expertise in the field of addiction for outpatient detox and/or medication management of mood disorders, anxiety disorders, or drug-induced psychotic disorders.
7. Weekly Individual psychotherapy with an expert in the field of addiction and certified in the AIT which includes CBT and mindfulness, to work on underlying emotional traumas and intrapersonal and interpersonal dynamics.
8. Neuromodulation as necessary.
9. Weekly individual counseling with a chemical dependency counselor to work on day-to-day addictive behavioral modification.
10. 12-step program attendance and participation by obtaining a sponsor/or being enrolled in an outpatient group program.
11. 12-step programs for family members.
12. Relapse prevention program with the long-term recovery groups.

### Prevention strategy

It has been proposed that one of the most influential biological determinants of susceptibility to substance dependency and addictive behaviors, whether drug-related or not, is brain development during gestation and childhood. Recent research by Vincent Felitti, principal investigator of historical research for Kaiser Permanente and the U.S. Centers for Disease Control, lends support to this notion, suggesting that childhood experiences of dependence are a primary contributor to addiction, rather than substance dependence alone. However, this perspective is not entirely accurate [336]. The concept of preaddiction, introduced in 1971, offers insight into the significance of preexisting vulnerabilities in predisposing individuals to both substance and behavioral addictions [289].

While the concept of prediction is intriguing, a critical examination of the theory proposed by McLellan et al. reveals several shortcomings. Yatan Pal Singh Balhara, from the All-India Institute of Medical Sciences (AIIMS), New Delhi, India’s National Drug Dependence Treatment Center, and the Department of Psychiatry, has scrutinized the operationalization of prediction proposed by McLellan et al. The authors advocate for the incorporation of “prediction” into the diagnostic categories of mild and moderate SUD according to the DSM-5 criteria. However, this approach presents challenges and limitations [289].

First, the DSM-5 introduced a significant shift in diagnosing substance-related disorders by omitting terms such as “abuse,” “dependence,” and “addiction.” Instead, severity of SUDs is assessed based on the number of diagnostic criteria met, without distinguishing between individuals with and without addiction. Even individuals with mild or moderate SUDs may exhibit characteristics associated with addiction, as defined by the DSM-5 criteria. Therefore, categorizing individuals as “preaddiction” based on mild or moderate SUD severity fails to adequately capture the clinical complexity of addiction.

Furthermore, the DSM-5 provides valid diagnostic criteria for mild and moderate-severity clinical presentations, which may still indicate the presence of addiction. Treatment interventions should be tailored based on the severity of clinical manifestations, regardless of whether all criteria for addiction are met. If the concept of “preaddiction” aims to facilitate appropriate interventions for individuals at risk of developing addiction, it is essential to identify them using criteria distinct from existing diagnostic categories [289].

To clarify, RDS can manifest from birth due to genetic antecedent risk, which may predispose individuals to a heightened susceptibility akin to preaddiction. Moreover, polymorphic DNA antecedents could exacerbate the risk of epigenetic alterations induced by subsequent SUD. We acknowledge that addictive behavioral seeking is a complex interplay of spiritual, neurobiological, and multifaceted psychological factors. We posit that, over time, addiction researchers and clinicians may define preaddiction through various assessments, such as administering the validated RDSQuestionnaire29 (RDSQ29), genetic risk profiling, a modified brain health assessment, or diagnostic categorization of mild to moderate SUD [337, 338].

In a comprehensive meta-analysis of depression, recent GWAS involving millions of participants have identified common comorbidities with SUD, highlighting crucial correlations with the expression of NEGR1 in the hypothalamus and DRD2 in the NAc [339]. Despite the increasing prevalence of SUD and neuropsychiatric disorders, routine objective brain evaluations are not yet standard practice. The objective of the standardized Brain Health Check (BHC) is to gather pertinent data for the treatment of clinical syndromes in psychiatric patients. The BHC would encompass several reliable, precise, cost-effective, and objective assessments across memory, attention, neuropsychiatry, and neurological imaging domains. Recommended tests for inclusion in the BHC comprise the Millon Clinical Multiaxial Inventory III (Neuropsychiatric), Quantitative Electroencephalogram/P300/Evoked Potential, CNS Vital Signs (Memory), Test of Variables of Attention (Attention), and over 36 years of diverse computerized and written-based assessments encompassing memory, attention, psychiatric, and neurological imaging (Neurological imaging). To diagnose and address reward dysregulation and mitigate the transgenerational epigenetic transmission of dopamine dysregulation, we advocate for ongoing research into the integration of a novel standard BHC coupled with qEEG/P300/Evoked Potentials and genetically guided precision modulation of “dopamine homeostasis” [340]. The concept of prediction may foster the development of therapies aimed at ameliorating neurotransmitter abnormalities and other early signs of addiction, as well as preventing addiction initiation altogether.

While incorporating the RDSQ29 may enhance early detection and stratification of prediction, the GARS test will continue to provide insights into potential DNA antecedents for all addictive behaviors, including OUD. As per Fagiolini et al. [341], RDS encompasses genetic, neurological, and psychological dimensions of compulsive, impulsive, and addictive behaviors. Data collected from two collegiate and university samples using the RDSQ29 indicated significant associations with impulsivity and sensory seeking. Exploratory and confirmatory factor analyses (EFA and CFA) on sample 1 (N = 1726) and confirmatory analysis on a separate sample (N = 253) demonstrated satisfactory fit indices. Construct validity analyses revealed a significant correlation between sensation-seeking and the RDS scale. The RDSQ29, developed through EFAs, comprises four subscales (lack of sexual satisfaction, activity, social concerns, and risk-seeking behavior), with preliminary evidence supporting its utility in evaluating the psychological and behavioral components of RDS. The ongoing refinement of the RDSQ29 by Blum’s laboratory in collaboration with the Hungarian research team aims to serve as an early-life risk assessment tool for prediction, facilitating the identification of potentially harmful pre-addictive or RDS behaviors through a simple pencil-and-paper index [137–342].

We conclude by proposing a complementary measure of “prediction” derived from RDS, which could further facilitate early detection, staging, and therapeutic intervention for this construct and its optimal characterization. The heuristic utility of our proposal will be evaluated based on its ability to encompass specific clinical, genetic, and therapeutic aspects of the prediction phenomenon. Further research is warranted to elucidate the distinctive features of RDS in addictive versus other psychiatric and medical conditions, as well as their interactions in comorbid states. Our central contention is that early genetic testing is paramount for identifying prediction or RDS in children [134, 135].

Donald Meichenbaum, in his book “Treating Individuals with Addictive Disorders,” posits that both opioid and dopamine circuits play pivotal roles in addiction. The dopamine system is particularly active during the initiation and maintenance of drug use and other addictive behaviors, serving as a linchpin for the reinforcing patterns of various substances, including alcohol, stimulants, opiates, nicotine, and cannabis. Desire, wanting, and yearning, all driving forces behind addictive behaviors, are intricately linked to dopamine function, extending to non-drug addictions as well [343]. Conversely, opioids primarily modulate the pleasure-reward components of addiction, whether intrinsic or extrinsic. These circuits, intertwined with dopamine pathways, constitute integral components of the limbic system, often referred to as the emotional brain, which processes a spectrum of emotions such as love, joy, pleasure, pain, wrath, and fear. Emotions, serving a fundamental survival function, regulating attachment, and aversion drives essential for human survival. While our emotional brain typically guides us toward nurturing experiences and away from threats, maladaptive coping mechanisms, including addiction, may arise when its function is compromised [344].

In infancy and early childhood, attachment bonds serve as pivotal environmental factors shaping brain development during periods of maximal growth, as outlined by Daniel Siegel in “The Developing Mind.” The immature brain leverages the adult functions of the parent’s brain through interpersonal connections fostered by attachment, with many reward gene polymorphisms predisposing individuals to addictive-like behaviors. These genetic antecedents, compounded by epigenetic influences, heighten susceptibility to both substance and behavioral addictions, contributing to a spectrum of psychological and behavioral issues such as anxiety, depression, and aggression. Children often mimic their parents’ behaviors and coping strategies, including addictive behaviors, possibly mediated by mirror neurons [345].

Moreover, recurrent exposure to early Adverse Childhood Experiences (ACEs), including physical, sexual, and emotional abuse, as well as neglect and community violence, escalates the risk of addiction. Individuals with four or more ACEs face a significantly heightened risk of alcohol and substance addiction. Traumatic childhood experiences can disrupt brain development, particularly in areas responsible for impulse control, leading to hypersensitivity to stimuli and experiences, as evidenced by phenomena like Rejection Sensitivity Dysphoria (RSD) observed in depression and potentially in ADHD [345].

Adolescent peer pressure can significantly influence the initiation of addictive behaviors. Beginning substance use or abuse in early adolescence within a peer group that also engages in substance use, including smoking, drinking, marijuana, and hard drugs, can lead to the brain becoming dependent on these substances. The earlier substance abuse begins, the higher the risk of developing addictive behaviors in adulthood [346].

Beneath maladaptive behaviors like addiction, a complex interplay of emotions such as fear, anxiety, shame, sadness, resentment, and anger often exist. When individuals lack the skills to manage and release these emotions effectively, they develop coping mechanisms to navigate daily life. The first instance of experiencing relief from overwhelming emotions through substance use ingrains a positive psychological association between the substance and a sense of survival and existence despite the drawbacks of real-life circumstances. This internal split gives rise to belief systems, values, behaviors, and emotions that may contradict logical reasoning [347].

Dr. Gabor Maté has identified anomalies in the Corpus Callosum (CC), a brain structure facilitating communication between the brain’s hemispheres, particularly among trauma survivors. Not only have trauma survivors been found to have smaller CCs, but evidence also suggests functional disturbances, potentially leading to a “split” in emotional processing. Stress may further exacerbate the disconnect between the brain’s two halves, hindering the integrated processing of positive and negative emotions [169]. Dr. Martin Teicher suggests that negative and positive feelings may be stored separately in different brain hemispheres, contributing to fragmented perceptions of oneself, others, and the world in intimate relationships and other life domains [348].

Substance addiction typically arises from the convergence of three factors: a susceptible individual, a substance with addictive properties, and stress. Individuals with heightened stress responsiveness may place a premium on substances or activities offering immediate relief while discounting long-term consequences. Exogenous opioid agonists, for example, modulate approach-oriented emotions like pleasure and anger while inhibiting avoidance-oriented emotions like fear and sadness. Prolonged opioid abuse can disrupt circuits involved in social bonding and emotion recognition, impacting overall emotional resilience [91].

Effective prevention of addiction hinges on emotional regulation and management. Cultivating awareness of thoughts, emotions, behaviors, and their implications is essential. The ability to identify, experience, understand, and release emotions is critical for emotional well-being. As the opioid crisis continues to claim lives, it is imperative to acknowledge the adverse impact of DNA polymorphic antecedents and epigenetic insults on children’s neurodevelopment. Understanding these challenges underscores the importance of implementing flexible BHC-ups in educational systems to address developmental short-circuits and mitigate high-risk genetic variations [349, 350].

### Anti-opioid vaccines

There is currently a notable interest in the development of anti-opioid medications, particularly in light of the ongoing public health crisis surrounding OUD and overdose, which has claimed over 100,000 lives in 2023 in the United States alone, primarily driven by fentanyl and its analogues. Immuno-therapeutics, including vaccines, have emerged as potential intervention strategies to complement existing opioid replacement therapies (ORT) and mitigate the incidence of OUD and opioid-related overdose. However, it is essential to acknowledge the limitations of immune therapeutic-based vaccines in clinical practice. These vaccines are specific to each targeted opioid moiety, meaning that a vaccine developed against one opioid, such as codeine, may not effectively block the effects of another opioid, such as heroin. Consequently, individuals dependent on opioids may potentially switch to different opioids, necessitating a multi-vaccination approach to achieve comprehensive protection [126].

Despite these challenges, recent studies have shown promising results in targeting structurally distinct fentanyl analogues. Baehr et al. compared various vaccines, including a lead conjugate vaccine (F1-CRM) and novel formulations incorporating haptens derived from alfentanil and acetyl fentanyl (F8, 9a, 9b, 10), to evaluate their efficacy against drug-induced pharmacological effects in rats. While no vaccine provided significant protection against alfentanil, the lead formulations effectively reduced the effects of fentanyl, sufentanil, and acetyl fentanyl, including antinociception, respiratory depression, and bradycardia. Importantly, vaccination with F1-CRM also reduced drug levels in the brains of rats challenged with lethal doses of fentanyl. These findings support further investigation of F1-CRM as a candidate vaccine against fentanyl and selected analogues, warranting expedited review by the FDA for approval to address the urgent need to reduce opioid-related deaths [351].

In light of these insights, several preventive measures are recommended, including parental education:

1. The self-development of each parent is to learn the skills of emotional regulation, healing their childhood traumas, and learning healthier emotional, behavioral, and social skills based on the AIT [297].
2. Creating a healthy and loving environment with their mate as a couple and as the creator of the loving and nurturing context for the family unit.
3. Conflict resolution and effective communication skills.
4. Fostering a healthy attachment bond with the children.
5. Engaging in a loving and nurturing matter while directing and teaching their children.
6. Developing a close, safe conversation with teens fosters self-esteem and self-confidence.

In general, there are several action items necessary to assist in preventing peer pressure to indulge in substance abuse, including:

1. Emotional intelligence curriculum for daycare/preschools and 1–12K – developing a curriculum based on the AIT [300] that fosters mental health by adapting skills and training children to learn to become aware of their thinking and feeling process that leads to their behavior. Teach children to notice, name, learn, and release emotions/feelings. Teach children to become assertive in communicating their needs.
2. Teacher training - developing a structured model based on the AIT [308] for teachers to utilize in their classrooms to foster mental and emotional health in the way they treat students as role model for the students to learn to treat each other (e.g., animated child’s book on the neurochemistry, neurogenetics, and epigenetics of the workings of the brain).
3. Mental and emotional health classes based on the AIT [296] for Junior high and High schoolers - developing psycho-educational classes for students and their parents to foster emotional regulation, clear communication, and better relationships with parents, as well as guidance for parents to set clear and healthy boundaries with appropriate monitoring and quality family time.
4. Mental and emotional health classes for college students – developing a psycho-educational curriculum based on the AIT [297] for first- and second-year students to include life skills, communication skills, and financial management skills to prepare them for adulthood and reduce the impact of stressors.
5. Emotional health and SUD evaluation - internal medicine physicians to address and evaluate the mental and emotional health of their patients as well as their substance use/abuse and dependency to give appropriate referrals to psychiatrists, psychotherapists, chemical dependency counselors, and AA or Narcotic Anonymous.
6. Incorporating a recently developed RDSQ29 scale would help identify patients experiencing abnormal psychological issues in addition to genetic risk testing [133].
7. Induction of pro-dopamine regulation to provide for a more balanced approach leading to “dopamine homeostasis” instead of blocking dopamine function as observed with some FDA MATS [228].
8. Incorporation of some dopamine-boosting holistic techniques. Without the induction of epigenetic brain repair, or “dopamine homeostasis,” any other methodology is doomed to fail with high relapse rates.
9. Mandatory drug urine screening, including a comprehensive analysis of reported drugs (CARD) of patients during treatment and recovery [352] for up to three years to monitor progress and ensure brain recovery [235].

## Discussion

### Focusing on preaddiction assessment

The number of opioid-related fatalities has surged since 2000, with over 500,000 deaths recorded globally, with the United States exhibiting the highest per-capita rate. Despite increased federal funding to address the opioid crisis, opioid overdoses remain a leading cause of mortality. While extensive neuroimaging research by NIDA and NIAAA scientists seeks to unravel the neurobiological mechanisms underlying opioid use and misuse, the FDA and CDC are working to mitigate overdose-induced premature deaths through improved pharmaceutical approvals and prescribing guidelines for MAT. However, the current OST approach, involving potent opioids like buprenorphine combined with naloxone, has been criticized for perpetuating addiction rather than offering effective treatment [337].

Efforts by the CDC to curb the overprescription of analgesics have not stemmed the rise in overdoses. Research utilizing emotion detection technology has highlighted the chronic reduction in affect induced by legally prescribed opioids, prompting calls for a more scientifically grounded approach to achieving “dopamine homeostasis” through non-pharmacological means [353]. Positive research on the clinical effects of potential pro-dopamine regulators like KB220/kb220Z underscores the need for tools like the GARS test to identify DNA gene reward risk antecedents and guide personalized treatment approaches [132, 150].

Embracing simple genetic testing to stratify individuals at risk of “preaddiction,” akin to the concept of RDS, could revolutionize addiction treatment paradigms. Similar to prediabetes in diabetes management, early detection and intervention strategies targeting preaddiction could yield significant improvements in treatment outcomes and halt the progression to full-blown addiction [354]. Hypodopaminergic at the mesolimbic brain reward circuitry and associated neurochemical abnormalities collectively known as RDS may provide valuable insights into the hedonistic derailments underlying addictive behaviors [27].

We propose that early genetic testing for addiction risk alleles could provide crucial insights for the parents and caregivers of these children before they are exposed to psychoactive substances. Variations in reward genes, including oxytocin and vasopressin, which regulate dopaminergic activity, may influence family dynamics, parenting styles, and attachment patterns. Studies have revealed regionally specific differences in responses to both food and drugs (as well as other non-substance addictive behaviors) based on either a “surfeit” or “deficit” model [355, 356]. It is widely acknowledged that adolescents and young adults grappling with addiction often experience emotional dysregulation, potentially impacting emotional circuits such as the amygdala, and may struggle to seek comfort and support from others, particularly their parents [135]. This dynamic significantly influences the initiation, progression, and recurrence of drug use. Consequently, addressing affect management should be a priority to facilitate sustained recovery.

Preventive measures, such as public health campaigns like “Say No and This is Your Brain on Drugs” have shown efficacy in reducing first-time drug use. However, identifying individuals with genetic predispositions for addiction remains a challenge, emphasizing the need for genetic addiction risk assessment tools akin to those used in diabetes management. In spite of arguing in favor of GWAS and subsequent polygenic scoring, we believe that the simpler candidate gene assessment approach could provide a genetic health risk pointing toward a liability for future substance and non-substance seeking behavior. Developing and implementing such tools could revolutionize addiction prevention and intervention strategies.

### Futuristic “Standard of Care” to attenuate OUD

**Figure 10:**
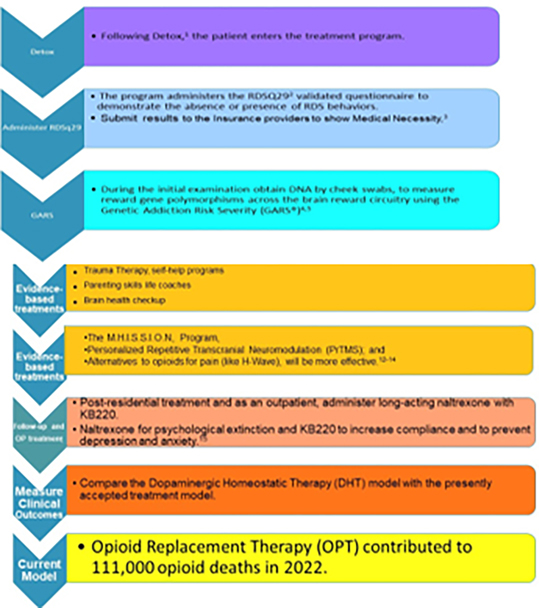
Dopaminergic homeostatic therapy (DHT) model.

Most recently, our laboratory published a detailed annotated bibliography of both the GARS and KKB220. In this document we illustrate that over the many years and variants of KB220 currently there are 35 peer reviewed trials in cluding triple and double-blind experiments, randomized treatment controls, demonstration studies, neuroimaging, genetic epigentic, and case studies [234]. Further research is required to encourage the field to consider (RDS) Anti-addiction Modeling” which involves early risk identification by means of genetic assessment similar to GARS (there are 89 PUBMED articles cited), followed by induction of dopamine homeostasis by means of genetically guided pro-dopamine regulation similar to KB220. These results suggest that genetically based treatments may be a missing piece in the treatment of SUD. In fact, we are proving a paradigm shift as depicted in figure 10.

## Summary

While the title of our paper may appear bold, it reflects a consensus among the co-authors, spurred by the urgency of addressing the ongoing failures and escalating opioid-related fatalities, particularly in the United States of America. Given this pressing need, significant paradigm shifts demand innovative thinking and approaches. In addition to advocating for further experimentation, we emphasize the importance of robust educational campaigns on a global scale through various media platforms, including social media, television, radio, and print media. These efforts aim to raise awareness about the concept of preaddiction, whether it manifests as a genetic trait or an epigenetic state, serving as a precursor to addiction, akin to the successful strategies employed in addressing prediabetes and tobacco addiction.

Moreover, it is imperative to continue rigorous investigations into the neurobiological, genetic, and neuro-epigenetic mechanisms underlying OUD table 3. With advancements in our understanding, particularly regarding how potent pharmaceuticals, especially in addiction psychiatry, contribute to undesirable neuroadaptations, there is hope that interventions such as genetic editing and gene therapy may one day offer preaddiction individuals a life free from the grips of addiction and filled with joy and vitality.

Crucially, transitioning to alternative non-addictive modalities, including genetic-guided interventions like KB220 (amino-acid-enkephalinase-N-acetylcysteine-NAD), non-invasive rTMS for psychiatry and pain management, epigenetic remodeling, gene editing, non-invasive H-wave therapy for pain relief and enhanced functionality, brain spotting, psychotherapy modalities such as AIT, CBT, mindfulness practices, trauma-informed therapy, genogram analysis, regular exercise, participation in sports, fitness programs (one hour per day), light therapy, and laughter therapy, among other known modalities capable of inducing reward symmetry, should be prioritized.

From our scientific standpoint, advocating for the reduction in the prescription of potent opioids, notably buprenorphine, and methadone, in favor of promoting the utilization of non-addictive alternatives like naltrexone alongside pro-dopamine regulation (e.g., KB220z) holds significant potential in emancipating individuals from the clutches of addiction and pain. This paradigm shift offers hope for restoring happiness to the approximately 2 billion individuals worldwide affected by RDS. This marks the advent of a transformative era where malfunctioning RNAs are potentially rectified through gene editing, guiding Homo sapiens toward a rejuvenated state. In alignment with this vision, our research team has recently undertaken an exhaustive pharmacogenomic analysis conducted in silico to enhance our comprehension of genetic pathways. This endeavor holds promise for offering unconventional solutions to pressing queries concerning the functionality of established neurotransmitters within the brain’s reward circuitry while identifying novel therapeutic targets (e.g., D3 polymorphisms).

A yet-to-be-published research study focusing on significant genetic mechanisms pertinent to opioids and pain conditions, including spine-related pain, delved into pre- and post-operative spine management and risk factors, scrutinizing 45 publications from 2008 to 2022. This comprehensive investigation employed a search strategy integrating pharmacogenomics (PGx) and spine pain as keywords to extract molecular evidence for subsequent bioinformatic analyses. The findings categorized three primary networks: Protein-protein interactions (PPIs), Protein-drug interactions (PDIs), and Gene-miRNA interactions (GMIs). PPI analyses unveiled a primary gene list (PGL) comprising 39 genes. Through PGx Variant annotation assessment, we reviewed 1,460 annotations, identifying 112 PGx-related variants, including 50 structural and regulatory variants and 62 intronic and non-coding variants. PDI results underscored specific compounds such as L-glutamic acid, Memantine, Haloperidol, and Ketamine, which are pivotal in linking pathways. GMI networks highlighted key microRNAs (miRNAs) potentially critical in spine pain management, including hsa-miR-16–5p, hsa-miR-199a-5p, hsa-miR143–3p, hsa-miR146a-5p, and hsa-miR144–3p. In a subsequent analysis, we conducted a novel PGx-based investigation of FDA-approved opioid medications in silico, titled “A PGx-based in Silico Investigation of Opioid Prescribing in Post-operative Spine Pain Management and Personalized Therapy.” This study identified 125 genes as targets of FDA-approved opioids, encompassing 7,019 variant annotations. Following in-depth filtration based on variant functions, 302 final filtered variants across 55 genes were identified. GMI analysis highlighted miR-16–5p as a pivotal miRNA in this network. PDIs revealed multiple drugs, including ibuprofen, nicotine, tramadol, haloperidol, ketamine, L-glutamic acid, caffeine, citalopram, and naloxone, with more than one interaction. Furthermore, PCIs highlighted key targets of the proposed chemicals, including ABCB1, BCL2, CYP1A2, KCNH2, PTGS2, and DRD2. Ten chemicals exhibited dual interactions with the target genes. This comprehensive review presents evidence-based, in silico findings regarding opioid prescribing in spine pain management, introducing 55 potential genes encoding FDA-approved receptors.

The insights from the *in-silico* report can serve as valuable guidance for exome analysis utilized as a PGx panel for assessing susceptibility to pain. This approach aids in tailoring individualized opioid prescriptions by genotyping pertinent variants. To achieve this, we delved into pain-related data housed within a comprehensively categorized PharmGKB database, orchestrating a PGx study centered on meticulously curated signaling pathways pertinent to pain, anti-inflammatory, and immunomodulating agents. This endeavor culminated in the identification of a gene panel comprising 128 genes. Subsequent filtration processes yielded 900 variants, among which 54 were deemed structural or regulatory variants of significance. Employing a spectrum of analytical methodologies including PPIs, Signaling Pathway Analyses (SPAs), GMIs, Transcription Factor-MiRNA Coregulatory Network analyses, PDIs, Pathway Connectivity Analyses (PCIs), and Genetic-Drug Association (GDA) assessments, we uncovered synergistic interactions among these 54 variants.

This study posits a novel approach for conducting pharmacogenomic Gene-Gene Interactions (GGIs) analyses of variants within personalized medicine frameworks, leveraging population-specific genomic data. Furthermore, it unveiled the Absorption, Distribution, Metabolism, and Excretion (ADME) properties of pain-related and opioid receptor genes, identifying six PGx variants associated with nine genes and seven opioids. Noteworthy associations include rs4292394 (UGT2B7) with methadone and lorazepam, rs2070959 (UGT1A6; UGT1A7; UGT1A8; UGT1A9; UGT1A10) with valproic acid, and rs12208357 (SLC22A1) with morphine, among others.

In an as-yet-unpublished investigation, advanced in silico analyses integrating SEPP1, exercise-induced Reactive Oxygen Species (ROS), aging, and Alzheimer’s disease were employed alongside PGx analyses. Drawing insights from the DisGeNET database, PDI modeling lent supportive evidence to the hypothesis under scrutiny. Of particular note, the Fruchterman-Rheingold model of PDIs underscored DRD3 as the principal gene, exhibiting high degrees of betweenness (degrees of betweenness = 4), with compelling PDIs bolstering this assertion. Noteworthy findings include associations of DRD3 with IL6 and TP35 in chronic alcoholic intoxication, alongside implications of DIO1 (a selenium target), DIO2, and DRD3 in thyroid diseases and bipolar disorders, hinting at potential connections to pathways involving exercise and ROS. Moreover, the study identified DRD3 and SEPSECS as critical players in seizures, shedding light on their possible roles in neurological disorders. Of significance, DRD2 and APOE emerged as contributors to PD, which aligns with our earlier discussions on neurodegenerative disorders. Additionally, associations were observed between amphetamine-related disorders (involving DRD4 and DRD3) and recurrent depression (involving DRD2 and DRD3), both categorized under addiction. Notably, these findings shed light on the role of D3 receptors in opioid dependence, as emphasized by Gondré-Lewis, who suggested that while opioidergic mechanisms contribute to OUD, dopamine-related receptors, particularly DRD3, may exert a primary influence on opioid-seeking behavior in African Americans. Consequently, developing novel and enhanced neuropharmacological therapies for OUD may necessitate focusing on DRD3-mediated regulation of dopaminergic homeostasis.

Recently and unfortunately, an article published in the New England Journal of Medicine concerning an NIH-funded (~400 million) intervention did not impact opioid-related overdose death rates over the evaluation period that was launched in 2019. The Healing Communities Study is the largest addiction prevention and treatment implementation study ever conducted and took place in 67 communities in Kentucky, Massachusetts, New York, and Ohio – four states that have been hard hit by the opioid crisis. The Healing Communities Study successfully engaged communities to select and implement hundreds of evidence-based strategies over the course of the intervention. Nora Volkow, the director of NIDA stated “Yet, particularly in the era of fentanyl and its increased mixture with psychostimulant drugs, it’s clear we need to continue developing new tools and approaches for addressing the overdose crisis. Ongoing analyses of the rich data from this study will be critical to guiding our efforts in the future.

## Conclusion

The multifactorial evidence presented in this paper underscores the significant role of genetic and epigenetic factors in addiction, highlighting the critical need for early detection. Since the 1990s, Blum’s groundbreaking discovery has elucidated the intricate interplay between DNA polymorphisms and epigenetics, revealing a balanced influence of nature vs. nurture in shaping developmental trajectories. Blum and colleagues have consistently demonstrated the presence of genetic predispositions, DNA markers, and epigenetic disruptions leading to deficient dopamine levels and “RDS.” These findings underscore the urgency of early detection and fundamental diagnostic strategies based on early developmental markers, genetic predisposition, and DNA structure.

To address the need for pre-assessment, we advocate for the development of a Comprehensive Early-Childhood Addiction Assessment Kit (CEAAK) comprising direct and observational measurement tools for medical professionals. These assessments should include Multi-Assessment Developmental Tests (MADT), GARS, Awareness Integration Emotional Assessment, temperament/personality trait questionnaires, parental observation questionnaires, and other early childhood measurement tools. The compilation of such assessments will enable the creation of a multisystemic treatment approach and nutrigenomics tailored to the early detection of behavioral addictive markers, which is essential for combating the global opioid addiction crisis.

Recognizing OUD as a complex brain disorder necessitates a multifaceted approach that integrates physiological, psychological, and spiritual dimensions. Frontline modalities benefit from incorporating alternatives such as personalized rTMS alongside psychotherapeutic interventions like AIT. Emerging scientific evidence supports the neurochemical benefits of religiosity and spirituality in reducing relapse rates, underscoring the importance of holistic treatment approaches table 3.

## Figures and Tables

**Table 1:** This chart includes attributable deaths for each drug; some involve multiple drug types, and percentage totals will exceed 100% [11]. https://drugabusestatistics.org/drug-overdose-deaths

Drugs Used in Overdose	%of Total Deaths
Opioid	67.80%
Cocaine	21.20%
Psychostimulants	20.60%
Methadone	4.03%

**Table 2:** Genes involved with pain mechanisms

Gene name	Polymorphism	Pathway(s)	Reference(s)
κ opioid receptor gene (OPRK1)	In humans, the 36G > T single nucleotide polymorphism (SNP) on KOR gene	The κ opioid receptor (KOR) system seems to play a role in stress responsivity, opiate withdrawal and responses to psycho stimulants, inhibiting mesolimbic dopamine. KOR gene polymorphisms have been reported to contribute to predisposition to voluntary alcohol-drinking behavior in experimental animals	[219]
Mu opioid receptor	A118G SNP of the mu opioid receptor gene (OPRM1)	Mu opioid receptors are critical for heroin dependence, and A118G SNP of the OPRM1 has been linked with heroin abuse. In our population of European Caucasians (n = 118), approximately 90% of 118G allelic carriers were heroin users	[220]
D(2) dopamine receptor gene (DRD2)	A haplotype block of 25.8 kb was defined by 8 SNPs extending from SNP3 (TaqIB) at the 5′ end to SNP10 site (TaqIA) located 10 kb distal to the 3′ end of the gene	Within this block, specific haplotype cluster A (carrying TaqIB1 allele) was associated with a high risk of heroin dependence in Chinese patients (P = 1.425 × 10(−22); odds ratio, 52.80; 95% confidence interval, 7.290–382.5 for 8-SNP analysis). A putative recombination ‘hot spot’ was found near SNP6 (intron 6 ins/del G), creating 2 new daughter haplotypes that were associated with a lower risk of heroin dependence in Germans (P = 1.94 × 10(−11) for 8-SNP analysis). Other studies show the relationship of carrying TAq1A1 vs A2 alleles in the treatment outcomes for heroin abuse. The results indicate that DRD2 variants are predictors of heroin use and subsequent methadone treatment outcome and suggest a pharmacogenetic approach to the treatment of opioid dependence. Others found an association between nasal inhalation of opiates and DRD2 promoter - 141 Delta C polymorphism. Significantly stronger cue-elicited heroin craving was found in individuals carrying DRD2 TaqI RFLP A1 allele than the non-carriers (P < 0.001)	[221–223]
ANKK1 gene	With a non-synonymous G to A transition, rs2734849 produces an amino acid change (arginine to histidine) in C-terminal ankyrin repeat domain of ANKK1	Since DRD2 expression is regulated by transcription factor NF-κB, we suspect that rs2734849 may indirectly affect DRD2 density. The rs273849 ANNK1 variant alters the expression level of NF-κB related genes	[224]
Catechol-O-methyltransferase (COMT) gene	Val (108/158) met polymorphism of the catechol-O-methyltransferase (COMT) gene	Genotyping 38 Israeli heroin addicts and both parents using a robust family-based haplotype relative risk (HRR) strategy. There is an excess of the Val COMT allele (likelihood ratio = 4.48, P = 0.03) and a trend for an excess of the Val/Val COMT genotype (likelihood ratio = 4.97, P = 0.08, 2 df) in the heroin addicts compared to the HRR control group	[225, 226]
Proenkephalin gene (PENK)	> or =81 bp allele	Among the subjects with opioid dependence, 66% carried the > or =81 bp allele compared with 40% of subjects with other types of substance abuse (χ2 = 11.31, p < 0.004) and 49% of controls (χ2 = 6.0, p < 0.015). These results are consistent with the role of the PENK gene in opioid dependence. In another study, heroin abuse was significantly associated with PENK polymorphic 3’ UTR dinucleotide (CA) repeats; 79% of subjects homozygous for the 79-bp allele were heroin abusers. Such individuals tended to express higher PENK mRNA than the 81-bp homozygotes, but PENK levels within the nucleus accumbens (NAc) shell were most strongly correlated to catecholamine-O-methyltransferase (COMT) genotype.	[227, 228]
Serotonin transporter (hSERT)	Homozygosity at hSERT (especially 10/10) was associated with early opiate addiction, while genotype 12/10 proved to be protective	Reward system pathway	[229, 230]
Dopamine transporter (DAT1)	In the case of DAT1, genotype 9/9 was associated with early opiate addiction. The combination of hSERT genotype 10/10 with DAT1 genotype 10/10 was shown to be a risk factor of opiate abuse under 16 years of age	Reward system pathway	[231]
Cannabinoid CB1 (brain) receptor gene (CNR1)	A microsatellite polymorphism (AAT)n at the cannabinoid CB1 (brain) receptor gene (CNR1) consists of 9 alleles. The number of i.e. drugs used was significantly greater for those carrying the > or ≥ or = 5 genotype than for other genotypes (P = 0.005)	Cannabinoid receptors in the modulation of dopamine and cannabinoid reward pathways	[231]

**Table 3:** represents a summary of high-level messaging related to solving the opioid crisis.

***State of what is:*** • In 2025, it is estimated that a tragic toll of 165,000 lives will be lost due to opioid-related fatalities. • According to a bipartisan report by the American Medical Association (AMA), overdose deaths incur an annual cost of US $1 trillion. • While many individuals find success in abstaining from addictive behaviors through treatment programs or participation in the 12-Step Program & Fellowship, these interventions are not universally effective. • Over 2 million individuals are reported to be members of Alcoholics Anonymous (AA). • Elucidating the molecular neurobiological foundations of each stage of the 12-Step Program provides insights into individuals’ diverse approaches toward recovery. • The Mesolimbic System, recognized as the brain’s reward center, governs feelings of well-being. • Chemical messengers such as dopamine, serotonin, enkephalins, glutamate, acetylcholine, GABA, cannabinoids, and GLP1 interact within the reward center to stimulate dopamine release in the Nucleus Accumbens (NAc). • Variations in genes, receptors, and metabolic pathways can impair the “Brain Reward Cascade,” leading to hypodopaminergia and promoting substance use and addictive behaviors. • Chronic opioid use can induce addiction, drug tolerance, neuroadaptation, hyperalgesia, and Reward Deficiency Syndrome (RDS) due to hypodopaminergia. • Epigenetic modifications, including changes to DNA and histones, play a crucial role in neurodevelopment and may influence susceptibility to addiction, particularly during adolescence. • Adolescent substance abuse and addiction risk factors include attention deficit hyperactivity disorder (ADHD) and depression. • The interplay of various polymorphic genes associated with neurotransmitters and second messengers regulates dopamine release in the NAc within the mesolimbic brain region. • Traumatic events and reduced gene expression via epigenetics can contribute to addiction. • Genetic variations may impact pain tolerance and sensitivity. • While FDA-approved opioids effectively reduce harm, chronic use, especially in individuals with high Genetic Addiction Risk Severity (GARS) scores, may increase addiction risk and overdose rates. • Depression shows significant co-morbidity with substance use disorder (SUD), with genome-wide association studies identifying therapeutic targets such as NEGR1 and DRD2. • The medical model recognizes addiction as a disease, emphasizing the importance of comprehensive treatment approaches. • Individuals with opioid use disorder (OUD) and a history of trauma face significant emotional pain, loss of control, and social disconnection. **Treatment Recommendations:** • Treatment aims to restore psycho-social-spiritual integration and balance. • Cognitive Behavioral Therapy (CBT) may not consistently produce significant outcomes in OUD treatment, highlighting the need for personalized approaches. • Awareness Integration Therapy (AIT) offers an evidence-based treatment option to integrate fractured aspects of the self and promote self-awareness and healing. • Neuroimaging and neurofeedback can help modify disrupted functional connectivity associated with addiction. • Personalized Repetitive Transcranial Magnetic Stimulation (rTMS) is being explored as a noninvasive treatment for substance use disorder. • Medical marijuana dispensaries have shown promise in reducing opioid prescriptions, but further research is needed to assess their safety and efficacy. • GARS testing could aid in identifying pre-addiction risk and vulnerability to opioid-induced addiction. • Non-addictive alternatives like Naltrexone, combined with pro-dopamine regulators like KB220, may enhance treatment compliance and efficacy. • H-wave therapy and personalized rTMS offer alternatives for pain management without powerful opioids. • Continued research into OUD’s neurobiological, genetic, and neuro-epigenetic mechanisms is crucial for developing effective interventions. • Genetic editing and gene therapy hold promise for preventing addiction and promoting well-being in susceptible individuals. **Recommendations:** • The current approach of providing opioids to combat opioid dependence is ineffective, necessitating a reevaluation of the “standard of care.” • Emphasis should be placed on inducing dopamine homeostasis through safe and non-addictive alternatives like KB220. • Comprehensive treatment approaches that address physiological, psychological, spiritual, and societal aspects of addiction, such as Awareness Integration Therapy, are recommended.

## Abbreviations

AIDS: Acquired Immunodeficiency Syndrome
AIT: Awareness Integration Therapy
ASAM: American Society of Addiction Medicine
COWS: Clinical Opioid Withdrawal Scale
DSM-5: Diagnostic and Statistical Manual of Mental Disorders, 5^th^ Edition
FDA: Food and Drug Administration
HIV: Human Immunodeficiency Virus
NSAIDs: Non-Steroidal Anti-Inflammatory Drugs
OBOT: Office-Based Opioid Treatment
OUD: Opioid Use Disorder
OOWS: Objective Opioid Withdrawal Scale
OTP: Opioid Treatment Program
SOWS: Subjective Opioid Withdrawal Scale
TB: Tuberculosis
UROD: Ultra-Rapid Opioid Detoxification
WM: Withdrawal Management
NAc: Nucleus Accumbens
CDC: Centers for Disease Control and Prevention
BRC: Brain Reward Cascade
SUD: Substance Use Disorder
PFC: Prefrontal Cortex
RDS: Reward Deficiency Syndrome
rTMS: Repetitive Transcranial Magnetic Stimulation
